# CRISPR/Cas9 Immunoengineering of Hoxb8-Immortalized Progenitor Cells for Revealing CCR7-Mediated Dendritic Cell Signaling and Migration Mechanisms *in vivo*

**DOI:** 10.3389/fimmu.2018.01949

**Published:** 2018-08-28

**Authors:** Swantje I. Hammerschmidt, Kathrin Werth, Michael Rothe, Melanie Galla, Marc Permanyer, Gwendolyn E. Patzer, Anja Bubke, David N. Frenk, Anton Selich, Lucas Lange, Axel Schambach, Berislav Bošnjak, Reinhold Förster

**Affiliations:** ^1^Institute of Immunology, Hannover Medical School, Hannover, Germany; ^2^Institute of Experimental Hematology, REBIRTH Cluster of Excellence, Hannover Medical School, Hannover, Germany

**Keywords:** CRISPR/Cas9, Hoxb8, immortalization, dendritic cells, migration, CCR7, calcium signaling

## Abstract

To present antigens to cognate T cells, dendritic cells (DCs) exploit the chemokine receptor CCR7 to travel from peripheral tissue via afferent lymphatic vessels to directly enter draining lymph nodes through the floor of the subcapsular sinus. Here, we combined unlimited proliferative capacity of conditionally Hoxb8-immortalized hematopoietic progenitor cells with CRISPR/Cas9 technology to create a powerful experimental system to investigate DC migration and function. Hematopoietic progenitor cells from the bone marrow of Cas9-transgenic mice were conditionally immortalized by lentiviral transduction introducing a doxycycline-regulated form of the transcription factor Hoxb8 (Cas9-Hoxb8 cells). These cells could be stably cultured for weeks in the presence of doxycycline and puromycin, allowing us to introduce additional genetic modifications applying CRISPR/Cas9 technology. Importantly, modified Cas9-Hoxb8 cells retained their potential to differentiate *in vitro* into myeloid cells, and GM-CSF-differentiated Cas9-Hoxb8 cells showed the classical phenotype of GM-CSF-differentiated bone marrow-derived dendritic cells. Following intralymphatic delivery Cas9-Hoxb8 DCs entered the lymph node in a CCR7-dependent manner. Finally, we used two-photon microscopy and imaged Cas9-Hoxb8 DCs that expressed the genetic Ca^2+^ sensor GCaMP6S to visualize in real-time chemokine-induced Ca^2+^ signaling of lymph-derived DCs entering the LN parenchyma. Altogether, our study not only allows mechanistic insights in DC migration *in vivo*, but also provides a platform for the immunoengineering of DCs that, in combination with two-photon imaging, can be exploited to further dissect DC dynamics *in vivo*.

## Introduction

A key feature of dendritic cells (DCs) is their capability to migrate and transport antigens from peripheral tissues to secondary immune organs, thus inducing tolerogenic and inflammatory immune responses (1, 2). This migration is mediated mainly by the interaction of the chemokines CCL19 and CCL21 with their receptor, CCR7, expressed on DCs (3). In peripheral tissues, haptotactic gradients of CCL21 secreted by lymphatic endothelial cells attract CCR7^+^ DCs toward lymphatic capillaries (4) where locally released CCL21 regulates their entry into the vessel lumen (5, 6). Within lymph capillaries, DCs actively follow CCL21 chemokine gradients crawling toward collecting vessels, in which they are passively transported by lymph flow toward the lymph node (LN) (7). Lymph delivers transported cells into the LN subcapsular sinus (SCS), a space between LN capsule and cortex, where lymphatic endothelial cells lining the SCS ceiling form CCL21 (and possibly CCL19) gradients crucial for direct DC transmigration through SCS floor toward the T cell zone (8, 9).

Although the CCL19/21-CCR7 axis is indisputably the main axis regulating DC migration, many questions regarding the mechanisms of DC migration and function remain unresolved and hamper development of novel therapeutic and vaccination strategies. For example, it would be crucial to establish the relative importance of several other chemokine receptors and their ligands implicated in the migration of DCs, including CX3CL1–CX3CR1 (10) as well as CXCL12-CXCR4 (11). Furthermore, it would be essential to directly compare deficiency of various receptors implicated in DC migration through tissue, such as C-type lectin receptor CLEC-2 (12), hyaluronan (13), or sphingosine 1-phosphate receptors (14). Moreover, it would be important to visualize contributions of divergent signaling cascades, including genes involved in calcium signaling and cytoskeletal organization, in various stages of DC migration (1, 15). Last of all, these studies would need to be done in complex three-dimensional environments, ideally *ex vivo* or *in vivo* (16).

The recent discovery and application of clustered, regularly interspaced, short palindromic repeats (CRISPR)/CRISPR-associated nuclease (Cas9) technology in eukaryotic cells presented a milestone for genome engineering due to its simplicity (17–20). Single guide RNA (sgRNA) screens in bone marrow (BM) cells from mouse strains that express *Streptococcus pyogenes* Cas9 have already identified genes involved in B cell activation and differentiation (21) and DC activation (22, 23). However, another level of the CRISPR/Cas9 technology can be achieved by its coupling to long-term *in vitro* hematopoietic progenitor cell lines. These hematopoietic precursors, transiently immortalized by retroviral transduction with an estrogen-inducible form of the transcription factor Hoxb8 (24), were recently used for further transduction with lentiviruses coding for Cas9 and guide RNAs (gRNAs) (25, 26). Grajkowska et al. used CRISPR/Cas9 to target E protein transcription factor TCF4 in either protein coding or enhancer region to decipher mechanisms by which isoform-specific TCF4 expression controls the development of plasmacytoid DCs (25). Leithner et al. used a similar system to target *Itgb2*, coding for integrin β2, and *Ccr7* and reported that the knockout cells are impaired in integrin-mediated adhesion to glass surfaces and migration toward CCL19 in 3D collagen gels, respectively (26).

Transduction with Cas9 expressing lentiviruses used in previous studies, however, requires antibiotic selection that is time consuming and might affect differentiation potential of transiently immortalized Hoxb8^+^ hematopoietic progenitor cells (25, 26). To circumvent that problem, we used bone marrow (BM) cells from a Cas9 expressing mouse strain (22) and lentivirally transduced them with an inducible form of the transcription factor *Hoxb8*, creating conditionally immortalized murine hematopoietic cells. These cells could be expanded for weeks in cell culture, providing sufficient time for their genetic engineering by successive transductions with lentiviral vectors encoding for sgRNAs, while at the same time retaining their potential for differentiation into DCs, macrophages or granulocytes. Our lentiviruses also coded for fluorescent proteins, allowing not only for the selection of successfully transduced cells with gene editing, but at the same time also facilitated their tracking *in vivo*. We used Cas9-Hoxb8-derived DCs to track CCR7-mediated DC migration and visualize CCR7-mediated calcium signals while entering LN via afferent lymphatics.

## Materials and methods

### Animals

Mice were bred at the Central Animal Facility at Hannover Medical School under specific pathogen-free conditions. The following mouse strains were used: C57BL/6J (as donors for the generation of immortalized progenitor cells), C57BL/6N (as recipients for Hoxb8 cell-derived dendritic cells), B6J.129(Cg)-Gt(ROSA)26Sortm1.1(CAG-cas9^*^,-EGFP)Fezh/J (designated here as Cas9 mice), B6-Tg(TcraTcrb)1100Mjb Ptprca-Pepcb/Boy (OT-I Ly5.1 mice), B6Cg-Tg(TcraTcrb)425Cbn/J (OT-II Ly5.1 mice). All experiments were conducted in accordance with the local animal welfare regulations reviewed and approved by the institutional review board and the “Niedersächsisches Landesamt für Verbraucherschutz und Lebensmittelsicherheit (LAVES).”

### Antibodies and staining reagents

Following antibodies and staining reagents were used in this study: Brilliant Violet 510 anti-mouse I-A/I-E (clone M5/114.15.2), PE-Cy7 anti-mouse CD11c (N418), APC rat IgG2c κ Isotype control (RTK4174), PerCP-Streptavidin, PerCP-Cy5.5 anti-mouse CD8α (53–6.7), PerCP anti-mouse CD4 (RM4-5), APC anti-mouse CD40 (3/23), APC anti-mouse CD80 (16-10A1), APC Armenian Hamster IgG Isotype control (HTK888), FITC anti-mouse MHCII/I-Ab (AF6-120.1), APC rat IgG2b κ Isotype control (RTK4530), PE-Cy7 anti-mouse CD11b (N418) (all from Biolegend), PE anti-mouse CD11b (M1/70), PE anti-mouse TCR Vα2 (B20.1) (all from Invitrogen), eF660 anti-mouse CD11b (M1/70), eF450 anti-mouse CD11b (M1/70), PE anti-mouse F4/80 (BM8), PE anti-rat IgG2a k-Isotype control (BR2a), APC anti-mouse CXCR4 (2B11), Alexa Fluor 488 anti-mouse Lyve-1 (ALY7), APC anti-mouse CD117/cKit (ACKα), PE anti-mouse CD135/Flt3 (A2F10), APC anti-mouse CD11c (N418), PE-Cy7 anti-mouse Ly-6A/E/Sca-1 (D7), biotin anti-mouse CD115/M-CSFR (AF598), APC anti-mouse Ly6C (HK1.4), PE-Cy7 anti-mouse CD45.1 (A20), biotin rat IgG2a κ Isotype control (eBR2a), PE rat IgG2b κ Isotype control (eB149/10H5), APC anti-mouse CD86 (GL1), APC rat IgG2a κ Isotype control (eBR2a), PE anti-mouse CD197 (CCR7) (4B12), APC anti-mouse CD197 (CCR7) (4B12), APC-eF780 anti-mouse Ly-6G/GR-1 (RB6-8C5; all from eBioscience), ATT0647 GFP-Booster (ChromoTek), FITC anti-mouse CD317 (PDCA-1; JF05-1C2.4.1) (Miltenyl Biotec), PE anti-mouse CD11c (HL3) (BD Biosciences), PE-Streptavidin and Cy5 anti-mouse B220 (TIB146; both homemade).

### Vector construction and viral particle production

Third generation lentiviral self-inactivating (SIN) vectors co-expressing respective sgRNAs and fluorescent marker genes were based on pLKO_TRC005 (Addgene) and constructed in the following manner. sgRNA transcripts are initiated from human RNA polymerase III promoter U6 (hU6) and expression of the fluorescent marker genes is driven by enhancer/promoter sequences of the spleen focus forming virus (SFFV). To facilitate cloning of gene targeting protospacer sequences into the vector backbone, a 105 bp-BsmBI-stuffer sequence was inserted downstream of hU6 and upstream of the sgRNA scaffold. Nuclear export and stabilization of the vector's mRNA transcripts were enhanced by incorporation of the post-transcriptional regulatory element of the Woodchuck hepatitis virus (PRE) (27, 28). pLKO5.hU6.sgRNA.BsmBI-Stuffer.SFFV.dTomato.PRE was generated by replacing the Tet2 protospacer sequence of pLKO5.hU6.sgRNA.Tet2.SFFV.dTomato.PRE with the BsmBI-Stuffer from pRRL.PPT.hU6.BsmBI-Stuffer.SF.SpCas9.T2A.dTomato.PRE.LoxP using restriction enzymes NdeI and EcoRI. pLKO5.hU6.sgRNA.BsmBI-Stuffer.SFFV.Cerulean.PRE and pLKO5.hU6.sgRNA.BsmBI-Stuffer.SFFV.eYFP.PRE were obtained by removal of dTomato from pLKO5.hU6.sgRNA.BsmBI-Stuffer.dTomato.PRE via AgeI and BsrgI and insertion of AgeI/BsrGI digested Cerulean or EGFP cDNAs from pRRL.PPT.SF.Cerulean.PRE and pRRL.PPT.PGK.eYFP.Tubulin.PRE (both kindly provided by Tobias Maetzig, Hannover Medical School, Hannover, Germany), respectively.

Protospacer sequences were designed with the online tools CRISPR Design (http://crispr.mit.edu/) (Zhang Lab, MIT, 2015) or CCTop (https://crispr.cos.uni-heidelberg.de/) (29). Generation of pLKO5.hU6.sgRNA.Ccr7.SFFV.dTomato.PRE is described elsewhere (30). Lentiviral vectors expressing sgRNAs targeting *Cxcr4* and *Trpml* were cloned as follows: designed sequences were ordered as oligonucleotides carrying BsmBI overhangs at their 5′-ends. After phosphorylation and annealing of the respective oligonucleotide pairs, the double-stranded protospacer DNA was incorporated into the BsmBI site of pLKO5.hU6.sgRNA.BsmBI-Stuffer.dTomato.PRE, pLKO5.hU6.sgRNA.BsmBI-Stuffer.Cerulean.PRE and/or pLKO5.hU6.sgRNA.BsmBI-Stuffer.eYFP.PRE according to Heckl et al. (31). The following gene targeting protospacer sequences were used: *Ccr7*, GTTTGGCGTCTACCTGTGTAA; *Cxcr4*, ACGTTTTCATCCCGGAAGCA; *Trpml1*, TGCCAGCGGTACTACCACCG (sgRNA 1) and GGGCTGGTGAGGTCCCCACC (sgRNA 3).

The lentiviral SIN vector RRL.PPT.T11. Hoxb8.hPGK.M2.P2A.Puro.pre (LV.T11.Hoxb8.Puro) was generated from construct pRRL.PPT.T11.MCS.PGK.M2.pre. We introduced a codon-optimized mouse Hoxb8 transgene (protein coding sequence see NCBI Reference Sequence: NP_034591.1) under the inducible T11 Tet promoter (32). Codon optimization was performed with the GeneArt algorithm (ThermoFisher). The reverse transactivator M2 is constitutively expressed from the human phosphoglyceratekinase (hPGK) promoter. For selection, we used the puromycin resistance gene 3′ of M2, fused by a P2A self-cleaving site (33, 34). Details are available on request.

Lentiviral particles were produced by transient transfection of 293T cells based on the calcium phosphate precipitation method assisted by 25 μM chloroquine (Calcium Phosphate Transfection Kit and chloroquine from Sigma-Aldrich). 293T packaging cells were con-transfected with 15 μg pcDNA3.gp.4xCTE (HIV-1 Gag-Pol) (35), 5 μg pRSV-Rev (kindly provided by T. Hope, Northwestern University, Chicago, IL, USA), 3 μg K73 pEcoEnv-IRES-puro (36) and 5 μg sgRNA-expressing vector. Supernatants were harvested 24 and 48 h after transfection and 100-fold concentrated by ultracentrifugation (16–18 h, 13,238 × g, 4°C). Viral pellets were resuspended in IMDM (Biochrom) supplemented with 10% FBS (PAA Laboratories), 1% penicillin-streptomycin, and 1% glutamine (Gibco) (designated as complete IMDM) and stored at −80°C.

The MLV-based gammaretroviral vector plasmid pRSF91.GCaMP6S.i2.dTomato.pre was generated in multiple steps. First, the GCaMP6F open reading frame of pGP.CMV.GCaMP6F (37) was amplified with primers 5′-ATCCGCTAGCGCTACCGGTCTCAGATCTCG-3′ (forward) and 5′-GGTATGGCGGATCCTGATCTAGAGTCGCGG-3′ (reverse), thereby introducing 5′ the AgeI and 3′ the BamHI restriction site. The GCaMP6F amplicon was subcloned into a shuttle vector and verified by sequencing. Next, GCaMP6F was excised via AgeI/BamHI (1,395 bp) and ligated with 346 bp (BamHI/BspmI) and 5,849 bp (BspmI/AgeI) fragments of pRSF91.i2.dTomato.pre to obtain pRSF91.GCaMP6F.i2.dTomato.pre. Subsequently, part of the GCaMP6F cDNA was replaced with relevant sequences of GCaMP6S via a 4-fragment ligation. For this purpose, pGP.CMV.GCaMP6S (37) was cut with NheI and NotI and the 1,321 bp GCaMP6S fragment was ligated with 1,307 bp (NotI/BsrGI), 4,427 bp (BsrGI/MscI) and 535bp (MscI/NheI) fragments from pRSF91.GCaMP6F.i2.dTomato.pre. The resulting vector plasmid pRSF91.GCaMP6S.i2.dTomato.pre was also verified by sequencing.

The production of ecotropic gammaretroviral RSF91.GCaMP6S.i2.dTomato.pre vector particles was conducted as previously described (38, 39). The supernatants were 50-fold concentrated via ultracentrifugation at 13,238 × g and 4°C for 16–20 h. Viral vector pellets were resuspended in StemSpan medium and stored at −80°C until further usage.

### Generation and cell culture of hoxb8 progenitor cell lines

Mouse hematopoietic stem and progenitor cells were isolated from C57BL/6J or Cas9 mice using a lineage cell depletion kit (Miltenyi). A total of 1 × 10^5^ cells were pre-stimulated for 48 h in StemSpan SFEM (Stem Cell Technologies) supplemented with rm-IL-3 (20 ng/μl), rm-SCF (50 ng/μl), rh-Flt-3L, rh-IL-11 (each 100 ng/μl; all cytokines from PeproTech), and 1% penicillin-streptomycin (PAN Biotech) in a 24-well suspension plate (Sarstedt). Cells were transduced with LV.T11.Hoxb8.Puro particles (2x MOI 2.5) two times on Retronectin (Takara Clontech) coated wells in medium containing 4 mg/ml protamine sulfate (Sigma-Aldrich). For expansion, we transferred cells to 48-well plates and changed the medium to complete IMDM supplemented with cytokines as described above (except rm-SCF: 100 instead of 50 ng/μl) in the presence or absence of 1 μg/ml doxycycline (Dox; Takara Clontech) and/or 0.3–1 μg/ml puromycin (Puro; Invivogen). On day 4 post transduction (pt), cultures were passaged to 6-well plates and the vector copy number (VCN) per cell was determined as previously described (40). Cultures were fed every 2–3 days and diluted on day 8, 15, and 38 pt to 1 × 10^6^ cells in 4 ml of medium. On day 22–26 pt, samples selected with puromycin and doxycycline were frozen with 2 × 10^6^ cells per aliquot in 90% FBS + 10% DMSO (Merck).

For transduction of Cas9-Hoxb8 cells, viral particles (MOI 4.5 of 50x concentrated GCaMP6S encoding particles and 100 μl of 100x concentrated sgRNA encoding particles) were bound to Retronectin (Takara Clontech)-coated 96-well plates (Sarstedt) by centrifugation (30 min−2 h, 2, 000 × g, 4°C). After removing the supernatant, Cas9-Hoxb8 cells resuspended in complete IMDM medium supplemented with rm-SCF, rh-IL-11, rh-Flt3L, rm-IL-3, Dox, Puro and polybrene (Sigma-Aldrich; 4 μg/ml) were added to the plate, centrifuged (45 min, 700 × g, 32°C) and incubated at 37°C. In some cases, cells were incubated 30 min prior to and during transduction with 10 μM cyclosporine A (Sigma-Aldrich). After 24 h, cells were washed and resuspended in fresh complete IMDM medium supplemented with rm-SCF, rh-IL-11, rh-Flt3L, rm-IL-3, Dox, and Puro.

### Generation of macrophages

Macrophages were generated *in vitro* based on a protocol described by Ho and Sly (41). Briefly, bone marrow cells were cultured overnight in complete IMDM. Non-adherent bone marrow cells were collected the next day. Hoxb8 cells or non-adherent bone marrow cells were then transferred to complete IMDM supplemented with 5 ng/ml rm-M-CSF (Immuno Tools) and 150 μM 1-thioglycerol (Sigma-Aldrich). After 6 days of M-CSF culture, Hoxb8 and bone marrow cells, which have not become adherent by then, were removed and the remaining adherent cells were further cultured in the presence of M-CSF and 1-thioglycerol until analysis on day 9.

### Generation of DCs

DCs were generated *in vitro* as described previously (3). Briefly, bone marrow cells or Hoxb8 cells were cultured for 9 days in RPMI medium (Gibco) supplemented with 10% FBS (PAA Laboratories), 1% penicillin-streptomycin, 1% glutamine (Gibco), 2-mercaptoethanol (Sigma), and cell culture supernatant from a GM-CSF producing cell line (5% final concentration). On day 8 of culture, aliquots of cells were collected and stained for the expression of markers specific for DC, macrophages or monocytes. For activation, cells were treated with lipopolysaccharide (LPS; 1 μg/ml; Sigma-Aldrich) at day 8 of culture for the remaining 16 h. In all cases, DC differentiation and maturation status was assessed by analysis of the CD11c and MHCII expression. For intralymphatic injection, GM-CSF-differentiated cells were selected based on cell size by fluorescence-activated cell sorting to enrich DCs and to remove dead cells and doublets. Sorting yielded a purity of 78–89% CD11c^+^MCHII^+^ cells.

To check for their potential to differentiate into conventional or plasmacytoid DC (cDCs and pDCs, respecitvely) BM cells or Hoxb8 cells were cultured for 9 days in RPMI medium (Gibco) supplemented with 10% FBS (PAA Laboratories), 1% penicillin-streptomycin, 1% glutamine (Gibco), 2-mercaptoethanol (Sigma), together with cell culture supernatant from a Flt3L producing cell line, as described previously (42). On day 8–9 of culture, cells were harvested and analyzed by flow cytometry.

### Dendritic cell-induced proliferation of T cells *in vitro*

DCs were generated as described above. During the final 16 h of culture, they were incubated in the presence of lipopolysaccharide (LPS; 1 μg/ml; Sigma-Aldrich) and chicken ovalbumin grade VI (200 μg/ml, Sigma-Aldrich). After being washed twice, 10^4^ DCs were co-cultured in round-bottom 96-well plates with 10^5^ eFluor 670-labeled CD8^+^ or CD4^+^ T cells isolated by magnetic cell separation (CD8α+ or CD4+ T Cell Isolation Kit mouse, Miltenyi) from spleens and lymph nodes of OT-I Ly5.1 or OT-II Ly5.1 mice, respectively. After 3 days of co-culture, T cell proliferation was determined by flow cytometry on LSR II (BD) and analyzed with FlowJo (TreeStar) v.7 and v.10.

### Transwell migration assay

10^5^ *in vitro* differentiated DCs were resuspended in 100 μl complete RPMI and loaded in collagen-coated transwells (Corning BV, 5 μm pore size) that were placed in 24-well plates containing 600 μl complete RPMI containing 0, 10, 100 or 200 ng/ml CCL21 (Peprotech). After incubation for 2 h at 37°C 5% CO_2_, migrated cells were collected and a defined number of 6 μm YG Fluoresbrite Microparticles (Polysciences) were added for counting of migrated cells by flow cytometry.

### Gene editing efficiency analysis

DNA was isolated from Hoxb8 cells using QIAmp DNA Mini Kit (Qiagen) and sgRNA target sites were amplified by PCR with NEBNext® High-Fidelity 2X PCR Master Mix (New England Biolabs). The following primers were used: Ccr7 exon3: 5′-TGTGCTTCTGCCAAGATGAG-3′, 5′-TCAGCCCAAGTCCTTGAAGA-3′; Mcoln1/Trpml1 exon 2: 5′-GGGAGATCAGAAAGGATAACATC-3′, 5′-ACTCATTGCACATGAAGTTCTC-3′; and Mcoln1/Trpml1 exon 4: 5′-ACCATTGCCTTCCGACATCT-3′, 5′-GGTGTGCAAGTGACAAGGTTA-3′. PCR reactions were purified using QIAquick PCR Purification Kit (Qiagen) for Sanger sequencing. The composition and frequency of insertions and deletions (INDELS) was analyzed using ICE software (Synthego; https://ice.synthego.com/#/) (43).

### Intralymphatic injection

Intralymphatic transfer of Hoxb8 cell-derived DCs with or without gene modifications was performed as described previously (9). Briefly, 4 × 10^4^ cells of a defined population were injected in 5 μl of PBS into the afferent lymphatic vessel of the popliteal LN. In comparative studies, a total of 8 × 10^4^ cells of a 1:1 mixture of two populations were injected. Popliteal LNs were subsequently analyzed using either two-photon microscopy or immunohistology (see below).

### Two-photon microscopy

LNs were explanted immediately after intralymphatic injection and glued into a custom-built perfusion chamber using tissue adhesive (Surgibond). During imaging, LNs were continuously superfused with prewarmed, oxygenated (95% O_2_ and 5% CO_2_) RPMI medium supplemented with 5 g/l Glucose (Sigma) as described earlier (44). Images were acquired with an upright Olympus BX51 microscope equipped with a W Plan-Apochromat 20x/1.0 DIC objective (Zeiss), TrimScope scanning unit (LaVision Biotech), and Mai Tai Titanium:sapphire pulsed infrared lasers (Spectra-Physics). For excitation of GFP and dTomato the laser was tuned to 920 nm. Time-lapse series were generated on a view field of 300 × 300 × 90–100 μm for up to 2 h with 15–17 images acquired per z-stack every 15–17 s. Imaging data was analyzed using Imaris 7.4–8.4 (Bitplane). A median filter was applied on all movies to reduce background noise. Tracking of cells was performed automatically based on the dTomato signal of the cells. Manual corrections were applied where required and dead or dying cells were excluded from the analysis. GCaMP6S mean intensity values (arbitrary units) were exported to Excel (Microsoft). To account for difference in GCaMP6S expression between cells and differences between the location of cells within LNs, normalized GCaMP6S values for each frame were calculated as a difference of GCaMP6S signal and mean GCaMP6S value for the subsequent 3 minutes (12 frames) of the same track. Finally, data points with GCaMP6S normalized value >1,000 arbitrary units were considered as Ca^2+^ signals (see also Figure 11B for details).

### Immunohistological analysis

Popliteal LNs were explanted 4 h after intralymphatic injection, fixed overnight in 2% paraformaldehyde (PFA; Carl Roth) plus 30% (vol/vol) sucrose, embedded in Tissue-Tek OCT (Tissue-Tek, Sakura), and frozen on dry ice. 8 μm thick cryosections were counterstained in TBS/T (Tris-buffered saline + 0.05% Tween 20) at room temperature using anti-Lyve-1 antibody and GFP booster. High-resolution composite images of whole LN sections were acquired with a Zeiss AxioScan Z1 (Plan-Apochromat objective: 10x/0.45 M27; camera: Axiocam 506 mono) or a Olympus BX61 (UPlanSApo objective: 10x/0.4; camera: F-View II), respectively. All pictures were contrast adjusted. Distribution of cells and distance measurements to the SCS were performed using Imaris and ImageJ as described earlier (8).

### Statistical analysis

All analyses were done with GraphPad Prism (v4 or v7). We calculated *p*-values using the non-parametric Mann–Whitney test or Fisher's exact test for comparison of two groups, or Kruskal–Wallis test with Dunn's test to compare multiple groups. All data are pooled from at least two independent experiments, as indicated below each figure, and *p* < 0.05 was considered statistically significant.

## Results

### Generation of cas9-hoxb8 cells

**Figure 1:**
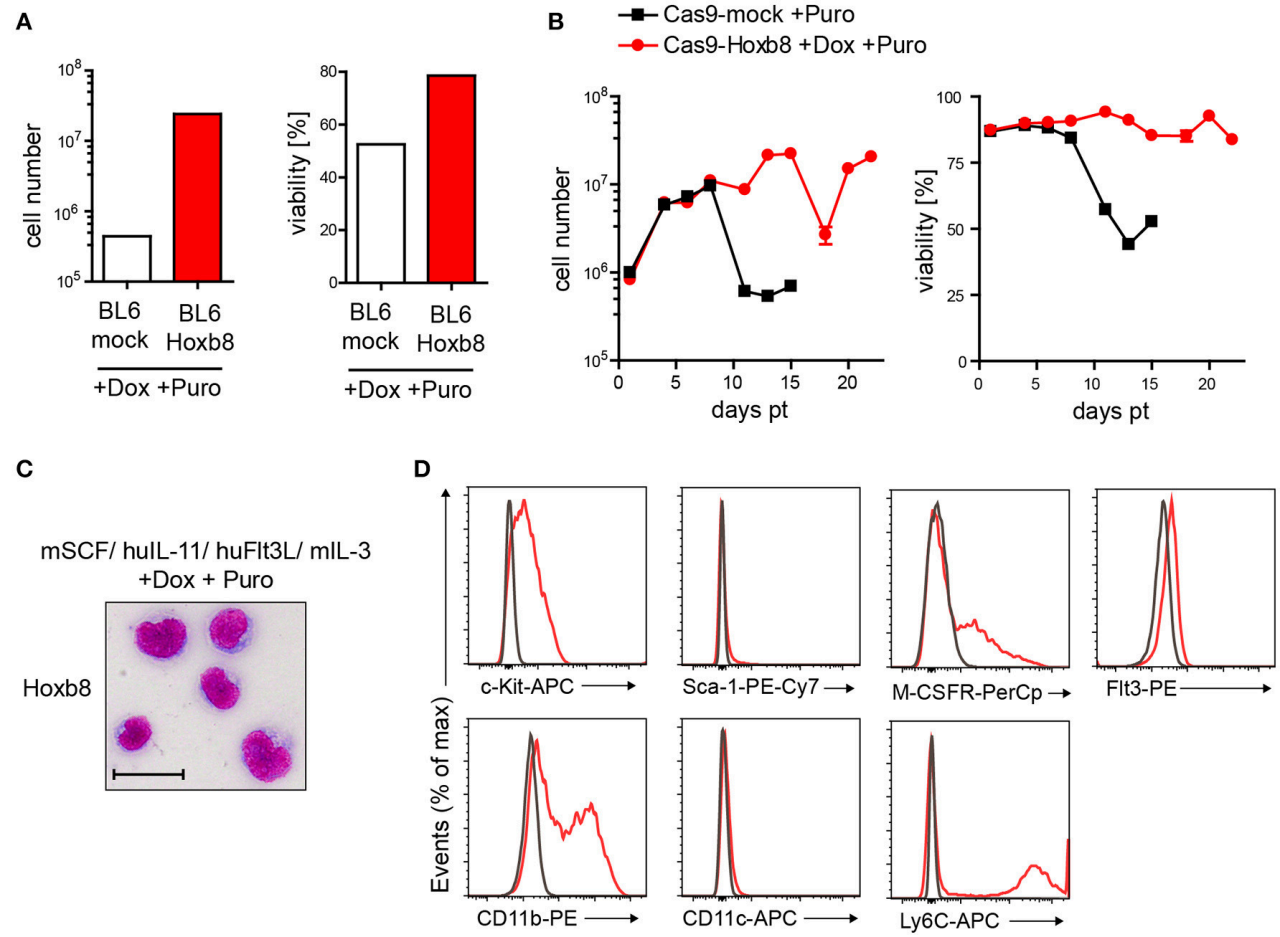
Immunoengineering of conditionally immortalized Hoxb8 cells from murine bone marrow. **(A,B)** Development of cell numbers and viability of lineage-negative BL6 **(A)** or Cas9 **(B)** bone marrow cells after mock infection or transduction with a Hoxb8-encoding lentivirus and subsequent culture in the presence or absence of doxycycline (Dox), and puromycin (Puro). **(A)** Cell numbers and viability from day 15 post transduction (pt) are shown. **(C)** Morphology (bright-field microscopy after cytospin and Pappenheim staining; scale bar: 25 μm) and **(D)** flow cytometric analysis of Hoxb8 cells grown in medium with mSCF, huIL-11, huFlt3L, mIL-3, doxycycline, and puromycin. Data are representative of two independent experiments **(C,D)**.

In contrast to previous studies, which used an estrogen-regulated form of Hoxb8 (24, 25, 26), we generated a doxycycline (Dox)-inducible third generation lentiviral vector for Hoxb8-mediated immortalization of murine hematopoietic cells. For enrichment of transgene positive cells, we also introduced a puromycin (Puro) selection cassette. To verify the vector-dependent immortalization of primitive hematopoietic stem and progenitor cells, we first transduced lineage-negative cells from C57BL/6J mice followed by cell culture in the presence of mSCF, huIL-11, huFlt3L, mIL-3, Dox, and Puro. Cells expressing the Hoxb8 construct were rescued from Puro treatment until day 15 post transduction (pt), whereas non-transduced cells showed low cell numbers and viability (Figure 1A). To allow for further genome modification with CRISPR/Cas9 technology, we isolated lineage-negative cells from Cas9 mice and selected them with the same strategy as described for the C57BL/6J mice. Only gene-modified cells survived longer than 15 days pt (Figure 1B). These cells, which we could stably culture for at least 16 weeks, were used for further experiments and were designated as Cas9-Hoxb8 cells.

Cas9-Hoxb8 cells showed a roundish cell shape (Figure 1C) and expressed c-Kit, while expression of Sca-1 was absent (Figure 1D), indicating that the cells are skewed toward the myeloid rather than lymphoid lineage. In addition, Cas9-Hoxb8 cells partially expressed CD11b, Ly6C and the M-CSF receptor, while CD11c could not be detected on their surface and Flt3 was only marginally expressed (Figure 1D). Low Flt3 expression did not result from receptor internalization as a consequence of culture in the presence of Flt3L, as Flt3L deprivation for 24 h did not result in Flt3 upregulation on the surface of Cas9-Hoxb8 cells (data not shown).

### Cas9-hoxb8 cells have the potential to *in vitro* differentiate into macrophages and dendritic cells

**Figure 2:**
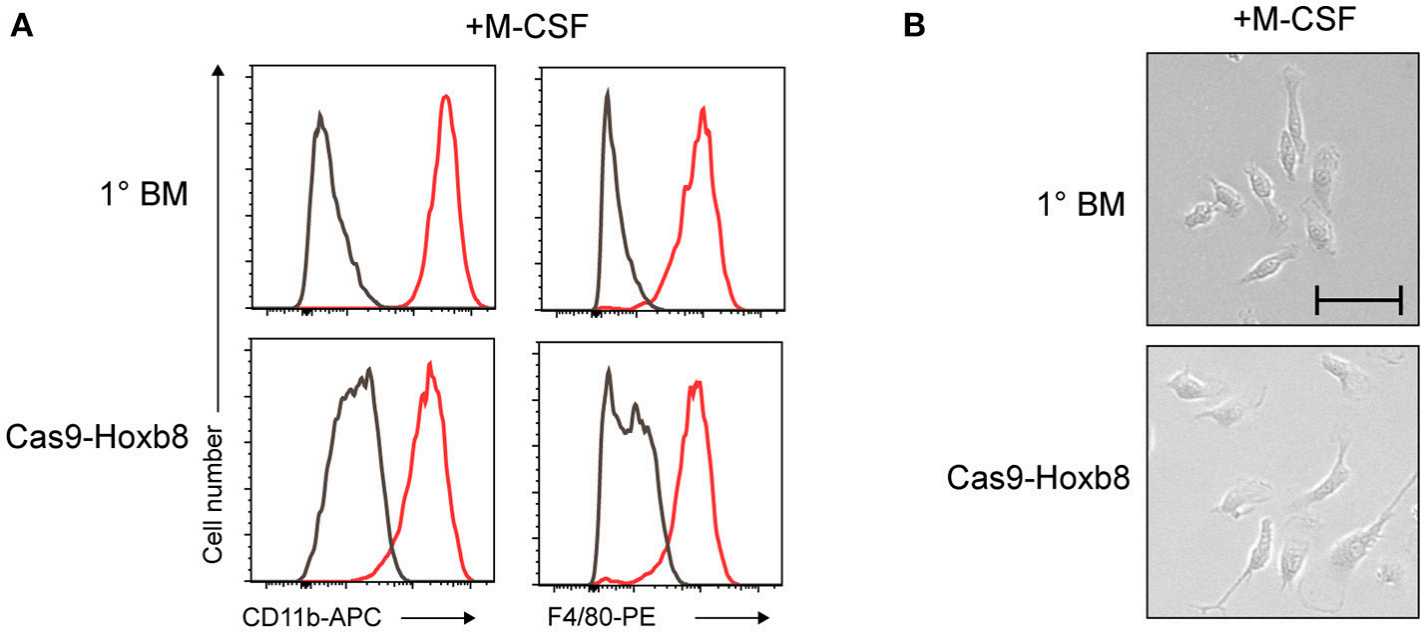
Phenotype of Cas9-Hoxb8 cell-derived macrophages. **(A)** Phenotype and **(B)** morphology (bright-field microscopy; scale bar: 50 μm) of primary BM cells (1° BM) or Cas9-Hoxb8 cells cultured in the presence of M-CSF for 9 days. Gray curves depict isotype controls. Data are representative of two independent experiments.

To further characterize the Cas9-Hoxb8 cells, we assessed their myeloid potential by withdrawing mSCF, huIL-11, huFlt3L, mIL-3, Dox, and Puro and exposing the cells to established *in vitro* myeloid cell differentiation protocols. First, the cells were cultured in the presence of macrophage colony-stimulating factor (M-CSF) according to a macrophage differentiation protocol described by Ho and Sly (41). On day 9 of M-CSF culture, Cas9-Hoxb8 cells exhibited expression of CD11b as well as F4/80 (Figure 2A) and the characteristic adherent morphology of macrophages (Figure 2B), just like macrophages derived from primary, freshly isolated bone marrow (1° BM) cells, which were treated according to the same protocol. The responsiveness of Cas9-Hoxb8 cells to M-CSF is consistent with their expression of the M-CSF receptor (Figure 1D).

**Figure 3:**
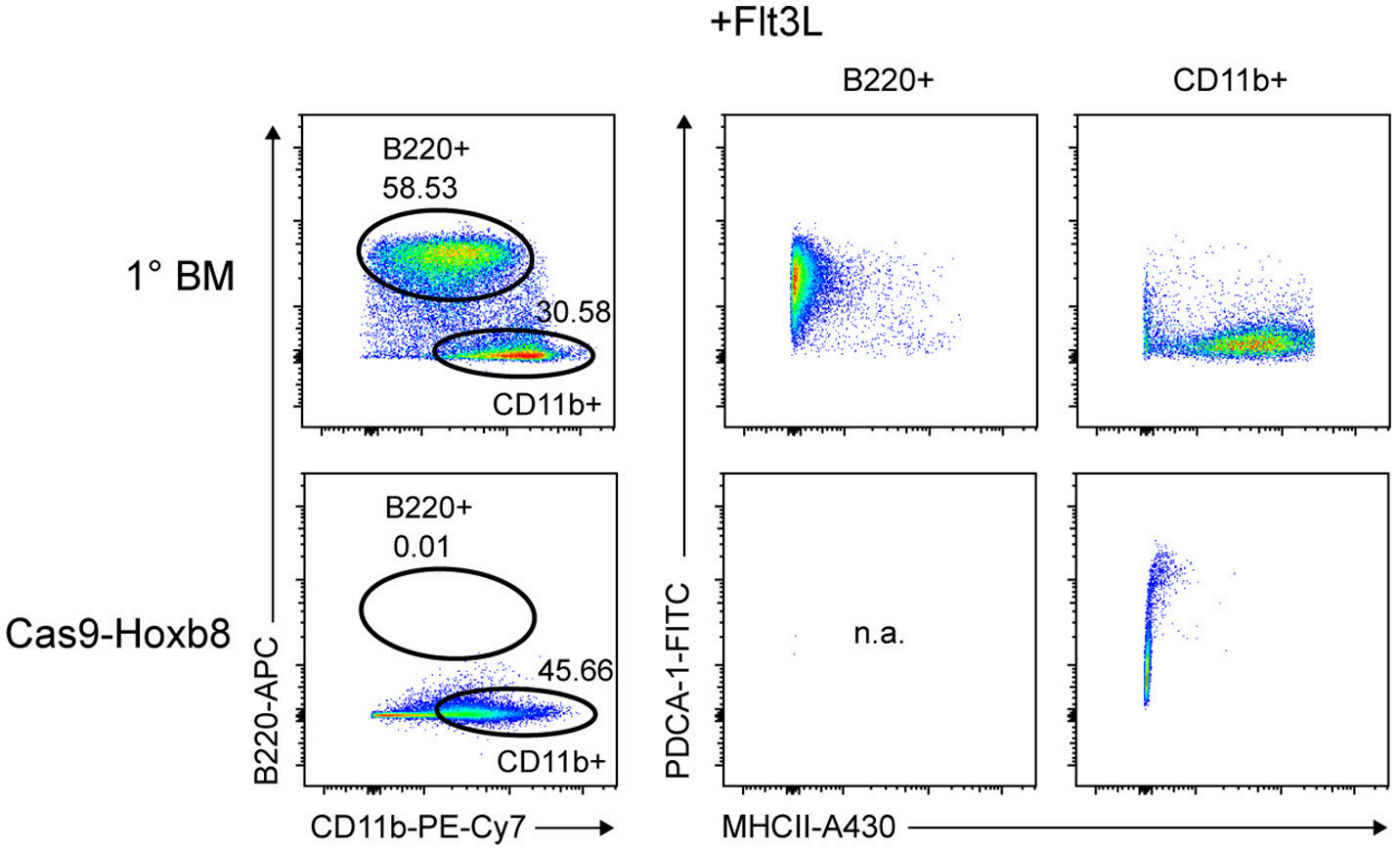
Cas9-Hoxb8 cells do not differentiate into plasmacytoid DCs in the presence of Flt3L.Flow cytometric analysis of primary BM cells (1° BM) or Cas9-Hoxb8 cells cultured for 8 days in the presence of Flt3L. In contrast to primary BM cells, Cas9-Hoxb8 cells failed to acquire a pDC phenotype (CD11b^−^PDCA-1^+^B220^+^). Data are representative for three independent experiments.

Next, we tested the differentiation potential of Cas9-Hoxb8 cells toward pDCs. Therefore we replaced mSCF, huIL-11, huFlt3L, mIL-3, Dox and Puro by Flt3L. Surprisingly—and in contrast to primary BM cells—Cas9-Hoxb8 cells failed to acquire a pDC phenotype (CD11b^−^PDCA-1^+^B220^+^; Figure 3). This unexpected observation is presumably due to the low expression of Flt3 on Cas9-HoxB8 cells (Figure 1D) impeding their Flt3L-driven differentiation into pDCs.

**Figure 4:**
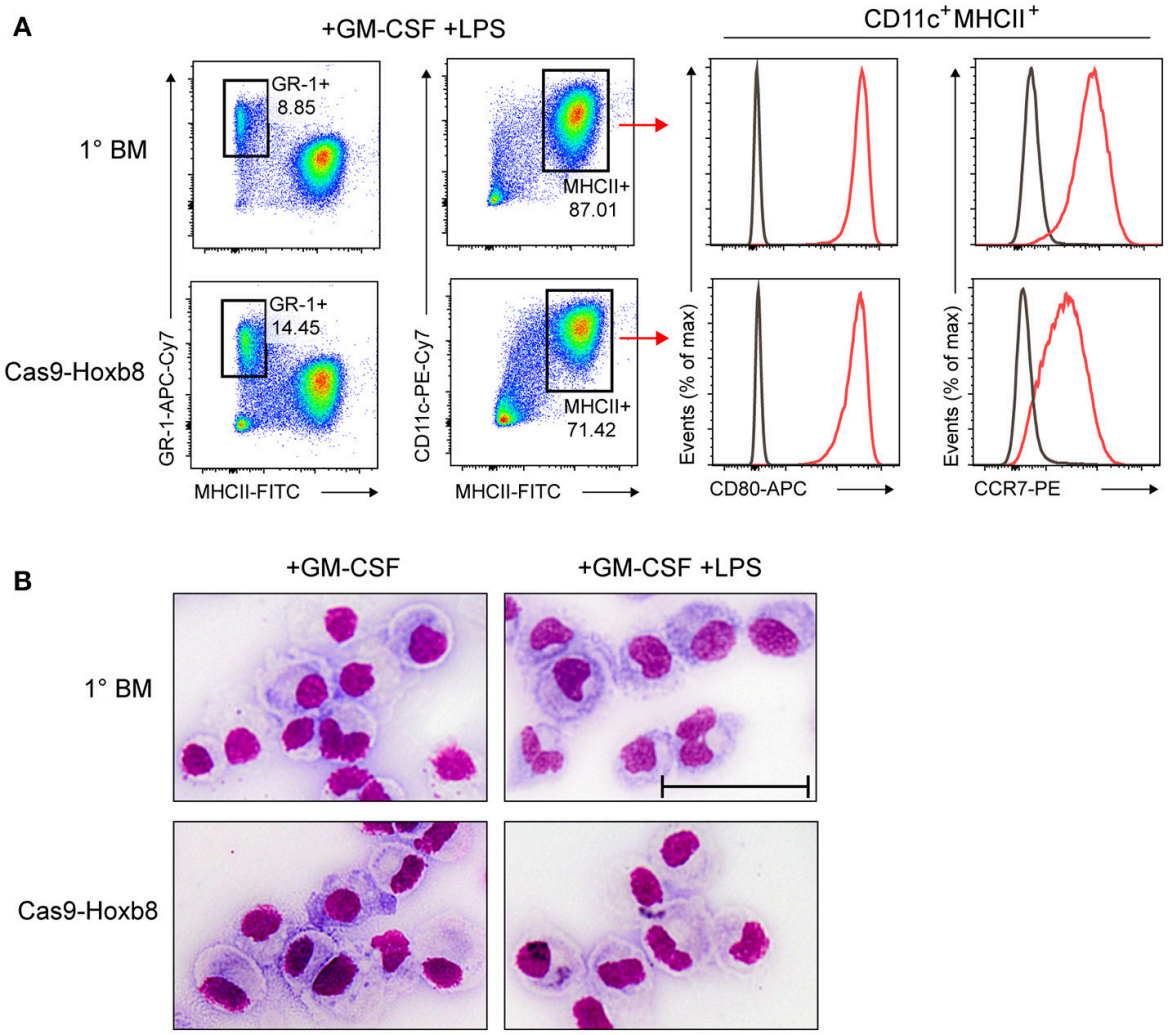
Phenotype of Cas9-Hoxb8 cell-derived DCs after LPS activation. **(A)** Flow cytometry analysis and **(B)** morphology (bright-field microscopy after cytospin and Pappenheim staining; scale bar: 50 μm) of primary BM cells (1° BM) or Cas9-Hoxb8 cells cultured in the presence of GM-CSF for 9 days followed by overnight treatment with LPS. Gray curves depict isotype controls. Data are representative of two independent experiments.

Next, we replaced mSCF, huIL-11, huFlt3L, mIL-3, Dox, and Puro with granulocyte-macrophage colony-stimulating factor (GM-CSF), which triggers the *in vitro* generation of dendritic cells and granulocytes. After 9 days of GM-CSF culture, cells were treated with the TLR4 agonist lipopolysaccharide (LPS) to induce DC maturation. GM-CSF-differentiated and LPS-treated Cas9-Hoxb8 cells showed a phenotype very similar to GM-CSF-treated and LPS-matured primary bone marrow cells, characterized by a major population of DCs (CD11c^+^MHCII^+^) and smaller population of granulocytes (GR-1^+^MHCII^−^) (Figure 4A). In addition, GM-CSF-differentiated and LPS-activated Cas9-Hoxb8 cells strongly up-regulated the co-stimulatory molecule CD80 and the chemokine receptor CCR7 (Figure 4A). Interestingly, although CD80 expression on Cas9-Hoxb8 cells was comparable to the level found on LPS-activated BM-DCs, CCR7 was expressed at slightly lower levels as compared to BM-DCs (Figure 4A). Further, Cas9-Hoxb8 cell-derived DCs acquired the typical morphology of BM-derived DCs (Figure 4B).

**Figure 5:**
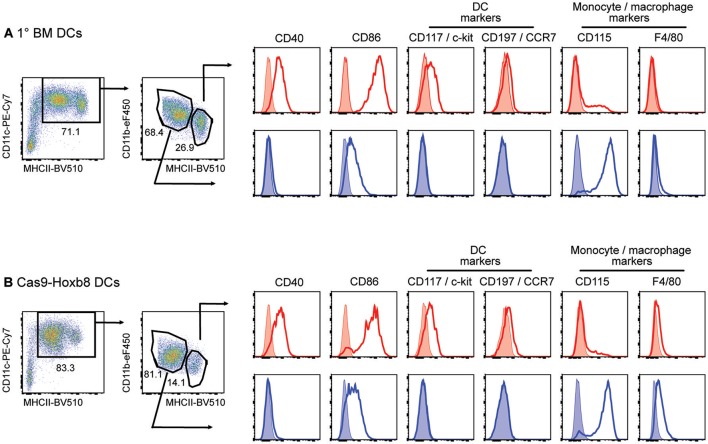
Phenotype of primary bone-marrow derived DCs (1° BM DCs) and Cas9-Hoxb8 DCs in CM-CSF cultures before LPS activation. Flow cytometric analysis of cells cultured for 8 days in the presence of GM-CSF. Without LPS activation, both **(A)** 1° BM and **(B)** Cas9-Hoxb8 cells were subdivided on the basis of CD11c, MHCII and CD11b expression. Histograms show surface expression of the markers indicated on MHCII^hi^CD11b^lo^ (red) and MCHII^int^CD11b^hi^ (blue) myeloid cell subsets. Data are representative for three independent experiments.

Interestingly, before LPS stimulation cells from both primary BM cells and Cas9-Hoxb8 cells express both macrophage and DC markers (Figure 5). This finding is in agreement with recent observations characterizing GM-CSF cultures of primary BM cells as a mixture of DCs and macrophages (45). In contrast, in our hands LPS activation of GM-CSF-differentiated Cas9-Hoxb8 DCs as well as BM-DCs creates population of cells showing homogenous expression of CD86 and CCR7 (Figures 4A, 7A). Since both markers are not expressed on BM-derived macrophages cultured in the presence of GM-CSF (45), we conclude that—similar to Leithner et al. (26)—Cas9-Hoxb8 cells differentiate into a relatively homogenous population of myeloid DCs.

In summary, Cas9-Hoxb8 cells possess the potential to *in vitro* differentiate into macrophages and dendritic cells.

### Cas9-hoxb8 cell-derived dendritic cells have the ability to induce T cell proliferation

**Figure 6:**
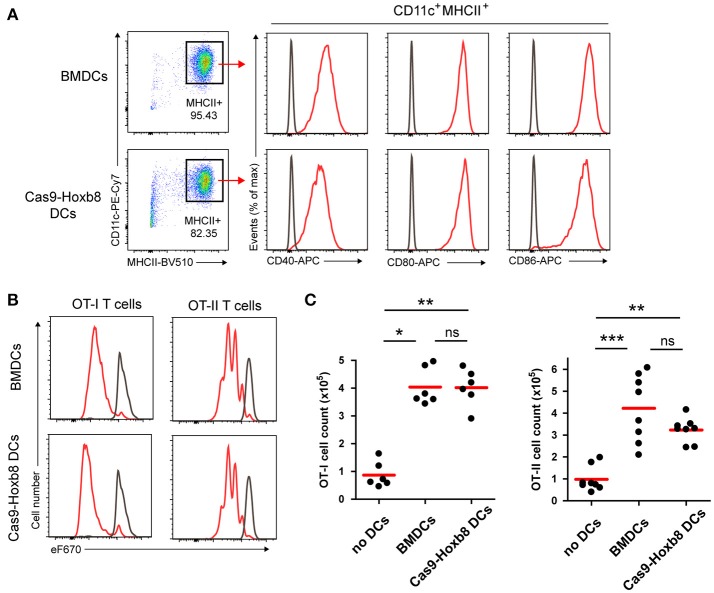
T cell activation potential of Cas9-Hoxb8 cell-derived DCs. **(A)** LPS-induced expression of the costimulatory molecules CD40, CD80, and CD86 on DCs derived from primary BM cells or from Cas9-Hoxb8 cells. Representative plots from three independent experiments. **(B)** Proliferation profiles of eFluor 670 (eF670)-labeled OT-I and OT-II cells cultured for 3 days with Ovalbumin (Ova) loaded, LPS-stimulated BM-derived or Cas9-Hox8 cell-derived DCs. Cells were gated as DAPI^−^CD45.1^+^CD8^+^ (OT-I) or DAPI^−^CD45.1^+^CD4^+^ (OT-II). Gray curves depict control profiles of OT-I and OT-II cells which were cultured in the absence of DCs. Representative plots from three independent experiments. **(C)** Numbers of OT-I and OT-II cells after co-culture for 3 days with ovalbumin loaded, LPS-stimulated BM-derived or Cas9-Hox8 cell-derived DCs. Data are pooled from three (OT-I) or four (OT-II) independent experiments. Red bars, mean; Kruskal–Wallis and Dunn's multiple comparisons test; ns, not significant; *p < 0.05; **p < 0.01; ***p < 0.001.

Given that GM-CSF-differentiated Cas9-Hoxb8 cell-derived DCs phenotypically resemble their BM-derived counterparts, we compared also their functionality for T cell activation. To that end, we loaded GM-CSF-differentiated Cas9-Hoxb8 as well as BM DCs with ovalbumin during LPS activation and mixed them with MHC class I or MHC class II restricted T cells carrying a transgenic T cell receptor specific for epitopes of ovalbumin. The cells are known as OT1 and OT2 cells, respectively and were stained with a proliferation dye prior use. In line with our previous observations, LPS-matured ovalbumin-loaded Cas9-Hoxb8 and BM-derived DCs showed an equally pronounced up-regulation of the co-stimulatory molecules CD40, CD80, and CD86 (Figure 6A). Consequently, Cas9-Hoxb8 cell-derived DCs induced a robust proliferation of CD8^+^ OT-I as well as CD4^+^ OT-II T cells comparable to that induced by BM-derived DCs (Figures 6B,C). Thus, Cas9-Hoxb8 cell-derived DCs are equally potent as BM-derived DCs in inducing T cell proliferation.

### Cas9-hoxb8 cells provide a source of genetically modified dendritic cells for the study of dendritic cell migration

**Figure 7:**
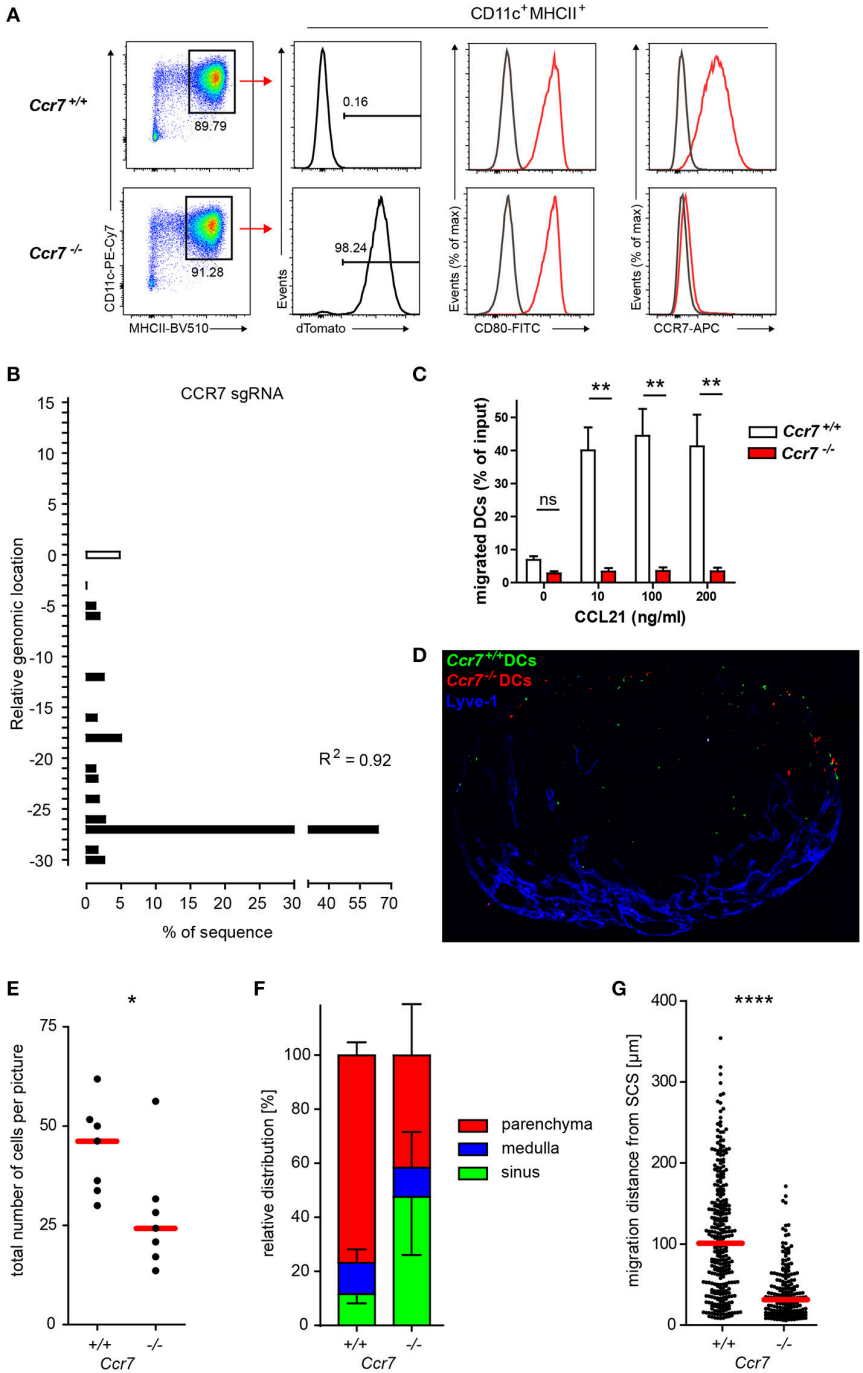
Phenotypic and functional verification of CRISPR/Cas9-mediated knockout of *Ccr7* in Hoxb8 cell-derived DCs. **(A)** Cas9-Hoxb8 cells were transduced with a dTom- and CCR7gRNA-encoding lentivirus. Successfully transduced cells were sorted based on the expression of dTom and subsequently differentiated to mature DCs in the presence of GM-CSF followed by treatment with LPS. *Ccr7*^+/+^ and *Ccr7*^−/−^ DCs were analyzed by flow cytometry for their expression of CD80 and CCR7. Data are representative for four independent experiments. **(B)** Analysis of the composition and frequency of insertions and deletions of *Ccr7*^−/−^ Cas9-Hoxb8 cells. R, Pearson correlation coefficient; *R*^2^ describes how strongly the calculated chromatograph of the indel distribution correlates with the Sanger sequencing results of the sample DNA. **(C)** Chemotactic migration of *Ccr7*^+/+^ and *Ccr7*^−/−^ DCs for 2 h toward medium alone or 10, 100, and 200 ng/ml CCL21. Data are pooled from 3 independent experiments with *n* = 8 in total. Mean + SEM; Kruskal–Wallis and Dunn's multiple comparisons test; ns, not significant; **p < 0.01. **(D)** Microscopy of popliteal lymph nodes obtained 4 h after intralymphatic injection of YFP-expressing *Ccr7*^+/+^ DCs and dTom-expressing *Ccr7*^−/−^ DCs (5–8 × 10^4^ cells in 5 μl PBS; scale bar: 200 μm). **(E)** Total cell counts and **(F)** relative distribution of *Ccr7*^+/+^ and *Ccr7*^−/−^ DCs 4 h after intralymphatic injection into popliteal LNs of B6 mice. **(G)** Migration distance from the subcapsular sinus (SCS) for the *Ccr7*^+/+^ and *Ccr7*^−/−^ DCs that entered LN parenchyma. Dots represent cell number per LN section **(E)** or individual cells **(G)**. Data are representative for **(D)** or pooled from **(E–G)** two independent experiments with a total of 7 lymph nodes analyzed. Error bars, SD; red bars, median; Mann–Whitney test; *p < 0.05; ****p < 0.0001.

In addition to T cell activation, migration is another key feature of DCs pivotal for their central role in immunity. For instance, to present antigens to cognate T cells, DCs travel from peripheral tissue to draining lymph nodes via afferent lymphatics in a CCR7-dependent manner (3, 9). To test if Cas9-Hoxb8 cell-derived DCs rely on the same mechanisms and thus can be exploited as a tool to study DC migration, we used lentiviral-based CRISPR/Cas9 technology to knockout *Ccr7*. Cas9-Hoxb8 cells were transduced with a lentivirus expressing dTomato (dTom) and sgRNA targeting *Ccr7* (30). Successfully transduced cells were purified by fluorescence-activated cell sorting based on dTom expression and subsequently differentiated into DCs in the presence of GM-CSF followed by the treatment with LPS. Flow cytometric analysis confirmed that dTom^+^ DCs completely lacked CCR7 expression despite being fully activated and exhibiting high levels of CD80 (Figure 7A). Interestingly, sequence trace decomposition that determines the composition and frequency of insertions and deletions indicated that 4.8% of cells still had intact sgRNA targeting locus (Figure 7B). Nevertheless, in contrast to dTom^−^ *Ccr7*^+/+^ DCs, these *Ccr7*^−/−^ DCs were not responsive to CCL21 in *in vitro* transwell migration assays (Figure 7C). Furthermore, 4 h after injection into the afferent lymphatics of popliteal lymph nodes, *Ccr7*^+/+^ DCs populated the lymph node T cell zone, whereas *Ccr7*^−/−^ DCs entered the lymph node parenchyma with delayed kinetics and failed to populate the deep T cell zone (Figure 7D), as it was observed in previous studies applying *Ccr7*-deficient BM-derived DCs in a similar setup (9). Quantification showed that there were significantly less *Ccr7*^−/−^ DCs than *Ccr7*^+/+^ DCs per picture taken (Figure 7E) and that almost half of *Ccr7*^−/−^ DCs was retained in the SCS, while more than 75% of *Ccr7*^+/+^ DCs penetrated into LN parenchyma (Figure 7F). Furthermore, *Ccr7*^+/+^ DCs that penetrated the LN parenchyma from the SCS floor progressed on average almost 4 times further toward the T cell zone than *Ccr7*^−/−^DCs (Figure 7G).

**Figure 8:**
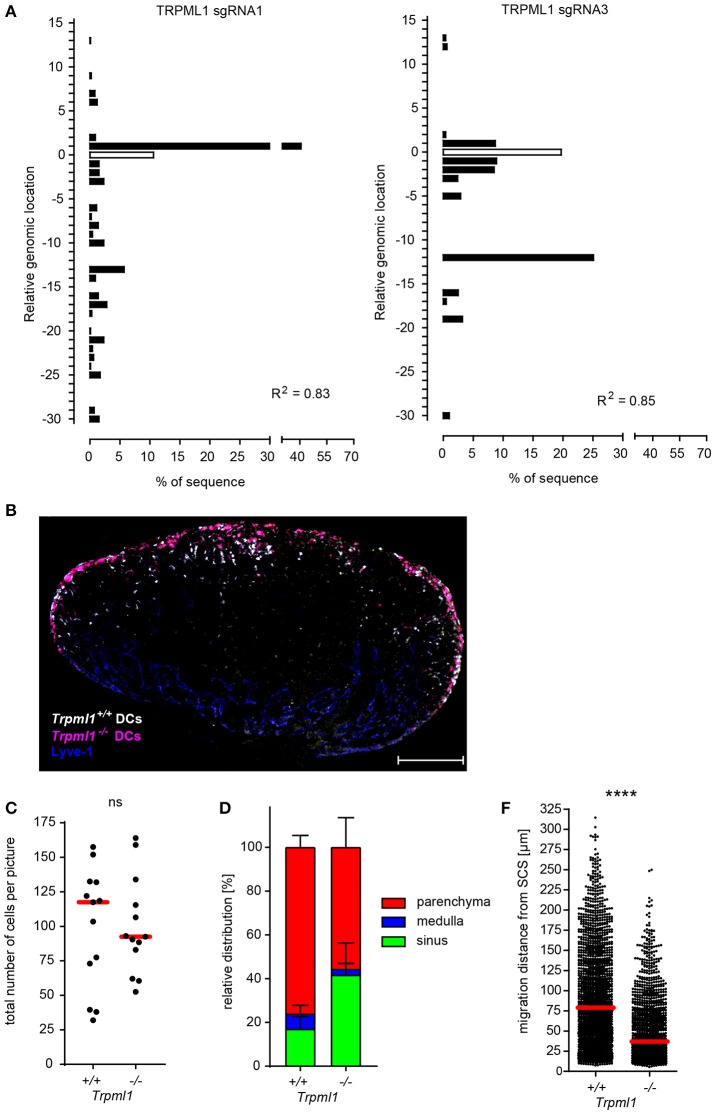
TRPML1 is required for efficient DC migration from the subcapsular sinus into the lymph node parenchyma. **(A)** Analysis of the composition and frequency of insertions and deletions of Cas9-Hoxb8 cells which were transduced with lentiviruses expressing different sgRNAs targeting *Trpml1*. Cas9-Hoxb8 cells which were transduced with lentiviruses expressing either sgRNA 1 or 3 were used to generate *Trpml1*^−/−^ DCs for further experiments. R, Pearson correlation coefficient; R^2^ describes how strongly the calculated chromatograph of the indel distribution correlates with the Sanger sequencing results of the sample DNA. **(B)** Microscopy of popliteal lymph nodes obtained 4 h after intralymphatic injection of YFP-expressing *Trpml1*^+/+^ DCs and dTom-expressing *Trpml1*^−/−^ DCs (1:1 mixture; 8 × 10^4^ cells in 5 μl PBS; scale bar: 200 μm). Data are representative for three independent experiments with 13 lymph nodes. **(C)** Total cell counts and **(D)** relative distribution of *Trpml1*^+/+^ and *Trpml1*^−/−^ DCs 4 h after intralymphatic injection into popliteal LNs of B6 mice. **(E)** Migration distance from the subcapsular sinus (SCS) for the *Trpml1*^+/+^ and *Trpml1*^−/−^ DCs that entered LN parenchyma. Dots represent average cell number per LN section **(C)** or individual cells **(E)**. Data are pooled from three independent experiments with a total of 13 lymph nodes analyzed. Error bars, SD; red bars, median; Mann–Whitney test; ns, not significant; ****p < 0.0001.

To further validate and expand our approach, we used lentiviral-based CRISPR/Cas9 technology to knockout *Trpml1*, a gene encoding for the ionic channel TRPML1 (transient receptor potential cation channel, mucolipin subfamily, member 1). TRPML1 was recently described to be required for fast and persistent migration of activated DCs, and TRPML1-deficient mature DCs migrated less efficiently to the draining LNs upon injection into the footpad (46). Due to the lack of TRPML1-specific antibodies for TRPML1 detection by flow cytometry, we confirmed *Trpml1* gene editing by sequence trace decomposition and found that two selected Trpml1-targeting sgRNAs (sgRNA 1 and 3) induce gene editing in more than 80% of the Cas9-Hoxb8 cells also expressing the fluorescent marker Cerulean encoded by the same lentivirus (Figure 8A), suggesting that the large majority, if not all, transduced cells has an edited *Trpml1* gene. Therefore, we used these cells as *Trpml1*^−/−^ Hoxb8-DCs and compared their migration during entry from afferent lymphatics into the lymph nodes via the SCS floor to *Trpml1*^+/+^ DCs (Figure 8B). We found slightly less *Trpml1*^−/−^ DCs compared to *Trpml1*^+/+^ DCs within the draining popliteal LN (Figures 8B,C). More pronounced was migration impairment of *Trpml1*^−/−^ DCs, preventing them from entry into the LN parenchyma and 4 h after i.l. transfer 41.6 ± 14.7% of these cells still resided in the LN sinus (Figures 8B,D). Furthermore, analysis of those cells that penetrated the LN parenchyma from the SCS floor revealed that, on average, *Trpml1*^+/+^ DCs had progressed more than 2 times further toward the T cell zone than *Trpml1*^−/−^ DCs (Figure 8E).

Collectively, these data suggest that Cas9-Hoxb8 cell-derived DCs rely on the same mechanisms for migration like BM-derived DCs and can therefore serve as an excellent tool to dissect DC migration.

### The unlimited proliferative capacity of cas9-hoxb8 cells allows consecutive genetic manipulations

**Figure 9:**
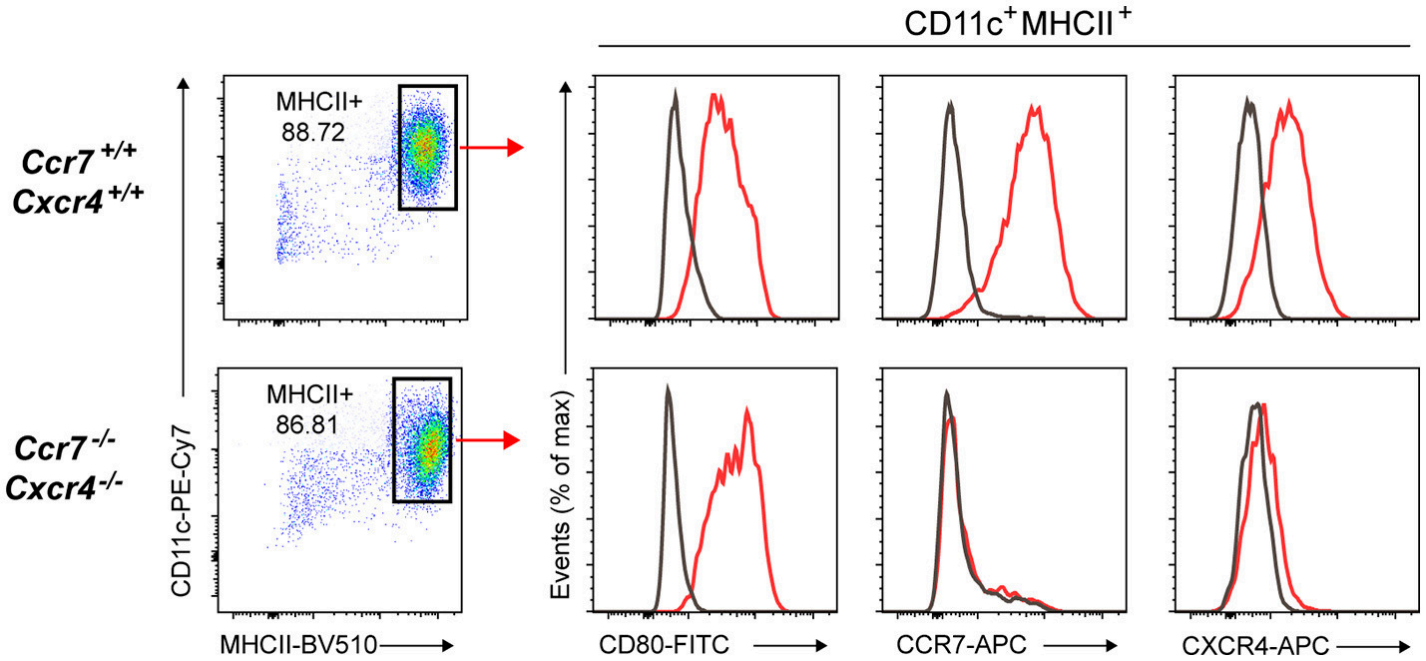
The unlimited proliferative capacity of Cas9-Hoxb8 cells allows the consecutive knockout of multiple genes.*Ccr7*^−/−^ Cas9-Hoxb8 cells were transduced with a Cerulean- and CXCR4-gRNA-encoding lentivirus. Transduced cells as well as control Cas9-Hoxb8 cells were subsequently differentiated to mature DCs in the presence of GM-CSF followed by the treatment with LPS. DCs were analyzed by flow cytometry for their expression of CD80, CCR7, and CXCR4. Gray curves depict isotype controls. Data are representative of two independent experiments.

The fact that Cas9-Hoxb8 cells could be maintained for a period of at least 16 weeks in the undifferentiated state while keeping full differentiation capacity offers many experimental benefits. Beside their potential for generating high numbers of knockout cells or their storage upon freezing for future experiments, the longevity also provides the opportunity to knockout multiple genes by consecutive genetic manipulations. To test this, we transduced dTom^+^ *Ccr7*^−/−^ Cas9-Hoxb8 cells with a lentivirus expressing Cerulean and a sgRNA targeting *Cxcr4*. This second round of transduction was done in the presence of cyclosporine A, as this has been demonstrated to enhance the transduction rate by overcoming a restriction against lentiviruses (47, 48). Transduced cells as well as control Cas9-Hoxb8 cells were subsequently differentiated into mature DCs. Flow cytometric analysis revealed that dTom^+^ Cerulean^+^ DCs lacked CCR7 expression and showed only marginal CXCR4 expression despite being fully activated and exhibiting high levels of CD80 (Figure 9).

### Cas9-hoxb8 cells also allow stable transduction with genetically encoded calcium indicator for tracking of chemokine-induced calcium signals *in vivo*

**Figure 10:**
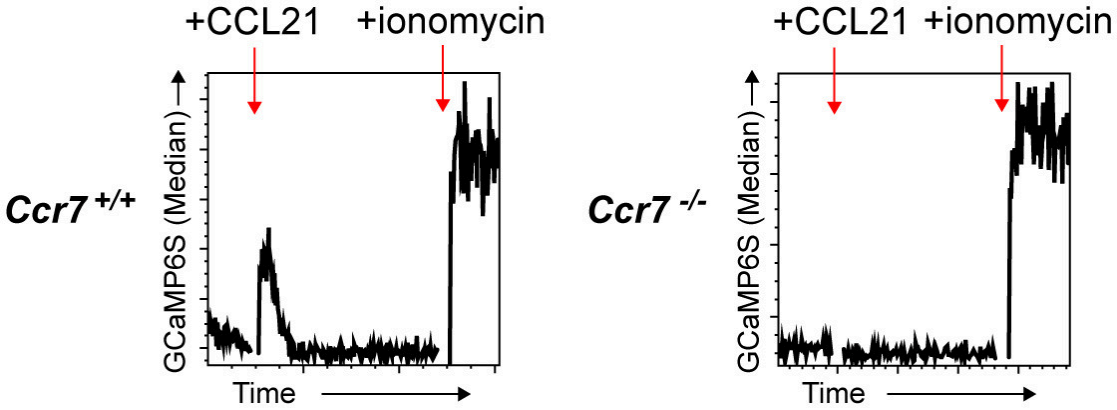
GCaMP6S functions as an efficient and specific sensor for Ca^2+^ flux in Cas9 Hoxb8 cell-derived DCs.*Ccr7*^+/+^ and *Ccr7*^−/−^ Cas9-Hoxb8 cells were transduced with a retrovirus expressing dTom and the Ca^2+^ sensor GCaMP6S. Successfully transduced cells were sorted based on the expression of dTom and subsequently differentiated to mature DCs in the presence of GM-CSF followed by LPS treatment. GCaMP6S intensity was measured by flow cytometry. After recording a baseline for 45 s, cells were treated with 500 ng/ml CCL21 and signals were recorded for additional 240 s. Finally, 1 μg/ml of ionomycin was added and signals were acquired for another 60 s. Data are representative for three independent experiments.

**Figure 11:**
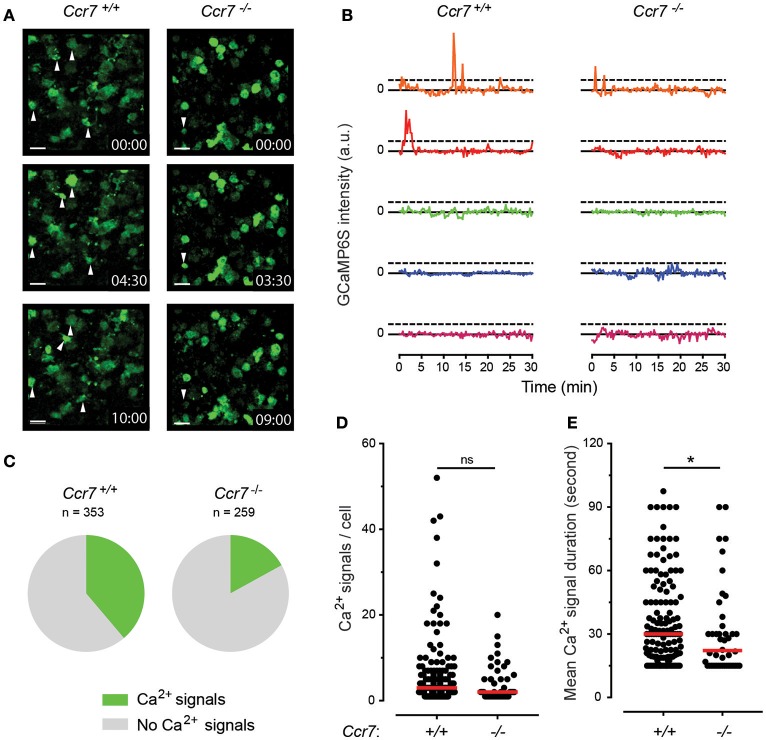
CCR7-induced Ca^2+^ signaling during entry of Cas9-Hoxb8-DCs transduced with Ca^2+^ sensor GCaMP6S into the lymph node. **(A)** *Ex vivo* time-lapse imaging of lymph nodes within 5 min after intralymphatic injection of 4 × 10^4^ *Ccr7*^+/+^ Hoxb8-DCs (left) or *Ccr7*^−/−^ Hoxb8-DCs (right) transduced with Ca^2+^ sensor GCaMP6S. White arrowheads indicate cells with changes in GCaMP6S signal intensity, indicating changes in Ca^2+^ concentration within the cell. For more details see Supplementary Video 1. Scale bar represents 15 μm. **(B)** Changes in GCaMP6S signal intensity for each 5 GCaMP6S^+^ *Ccr7*^+/+^ Cas9-Hoxb8-DCs and GCaMP6S^+^ Ccr7^−/−^ Cas9-Hoxb8-DCs. Data are represented as a difference in GCaMP6S signal intensity for each time point to the median value for next 3 min. Horizontal dashed lines depict thresholds [defined as a change in a signal intensity >1,000 arbitrary units (a.u.)] used to detect Ca^2+^ signals. **(C)** Pie-charts indicating the percentage of GCaMP6S^+^ *Ccr7*^+/+^ Cas9-Hoxb8-DCs or GCaMP6S^+^ Ccr7^−/−^ Cas9-Hoxb8-DCs with changes in Ca^2+^ signals. There is a significant difference between groups (*p* < 0.001, two-tailed Fisher's exact test). **(D)** Number and **(E)** average duration of Ca^2+^ signals (changes in GCaMP6S intensity) per cell for the tracks with at least one recorded Ca^2+^ signal. In **(D,E)** dots represent individual cells and red line median group value. Asterisk (*) indicates significant difference (*p* < 0.05), while ns indicates no significant difference (Mann–Whitney test). Data are representative **(A,B)** or pool **(C–E)** of 6 independent experiments with total of 7 lymph nodes per cell type.

To exploit the full potential of the multiple genetic modifications in Cas9-Hoxb8-DCs for investigating DC migration, we decided to retrovirally engineer *Ccr7*^+/+^ and *Ccr7*^−/−^ Cas9-Hoxb8-DCs to express the genetic calcium (Ca^2+^) sensor GCaMP6S (37) together with constitutively expressed dTomato. By this approach we aimed to create DCs proficient or deficient for CCR7 allowing recording Ca^2+^ signaling in real-time during DC entry into the LN parenchyma. Initial calcium flux assays using flow cytometry indicated that CCL21 specifically triggers calcium flux in *Ccr7*^+/+^ but not *Ccr7*^−/−^ Cas9-Hoxb8-DCs (Figure 10). We next intralymphatically injected GCaMP6S^+^ *Ccr7*^+/+^ or *Ccr7*^−/−^ Cas9-Hoxb8-DCs and imaged them by two-photon microscopy during the first 2 h after injection. Within this time frame, intralymphatically injected DCs are known to enter the lymph node parenchyma (9). Using this approach, we observed that many GCaMP6S^+^ *Ccr7*^+/+^ Cas9-Hoxb8-DCs exhibit prominent changes in GCaMP6S intensity during the recording period, while only few GCaMP6S^+^ *Ccr7*^−/−^ DCs showed signal alteration above background (Figure 11A and Supplementary Video 1). To quantify the Ca^2+^ signals, we tracked the injected cells based on their expression of the reporter gene dTomato (not shown) and analyzed mean intensity values of GCaMP6S intensity as described in Materials and Methods. Figure 11B illustrates differences in GCaMP6S intensity of representative GCaMP6S^+^ *Ccr7*^+/+^ or *Ccr7*^−/−^ Cas9-Hoxb8-DCs. Quantitative analysis revealed that 39% of injected GCaMP6S^+^ *Ccr7*^+/+^, but only 17% of GCaMP6S^+^ *Ccr7*^−/−^ Cas9-Hoxb8-DCs had at least one prominent change in GCaMP6S signal, indicating change in intracellular Ca^2+^ concentration (Figure 11C). A more detailed analysis of cells with changes in GCaMP6S signals indicated that there is no difference in the median number of calcium signals per GCaMP6S^+^ *Ccr7*^+/+^ and *Ccr7*^−/−^ Cas9-Hoxb8-DCs (Figure 11D), but that the average signal duration is significantly prolonged in cells expressing CCR7 compared to those were *Ccr7* had been disrupted (Figure 11E). Overall, these results suggest that Cas9-Hoxb8-DCs expressing the Ca^2+^ indicator GCaMP6S allow real-time tracking of Ca^2+^ signals during migration and chemokine recognition *in vivo*.

## Discussion

**Figure 12:**
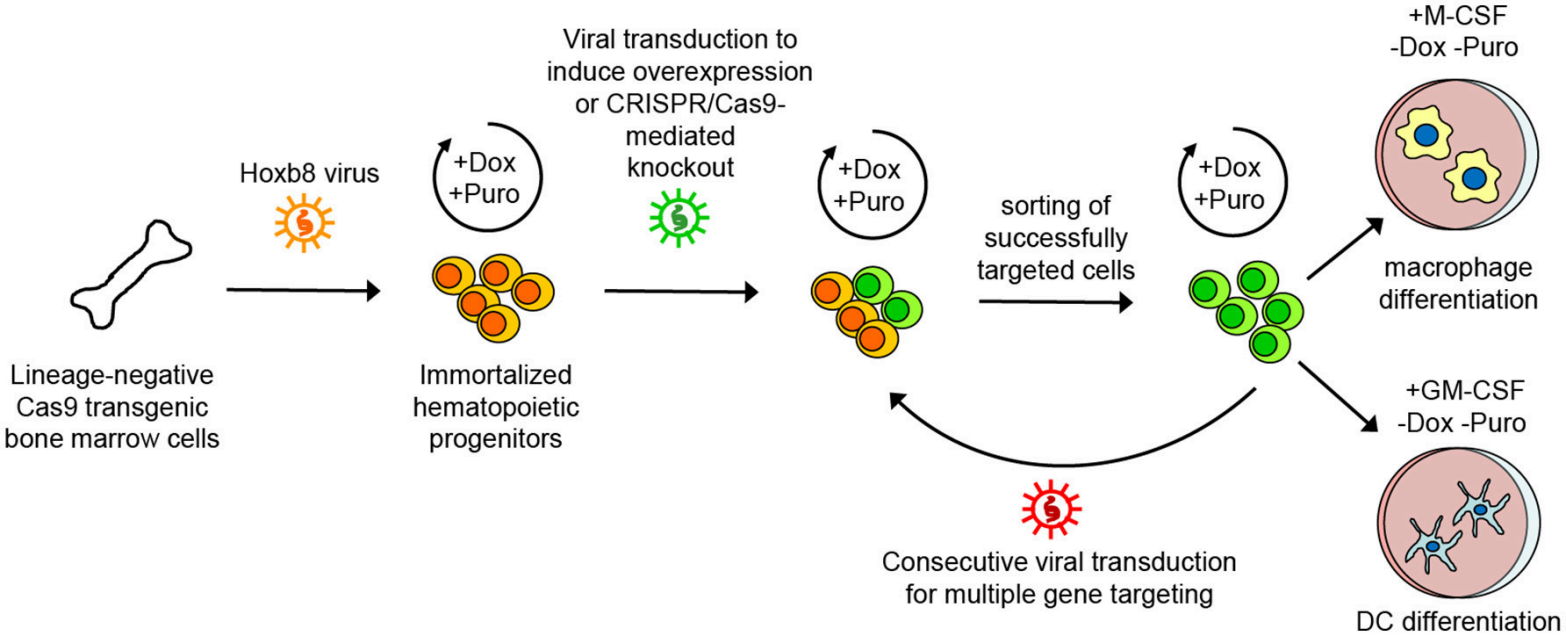
Scheme of generation, maintenance, genetic modification and differentiation of Cas9-Hoxb8 cells. Lineage-negative cells from the bone marrow of Cas9 transgenic mice were conditionally immortalized by lentiviral transduction introducing a doxycycline (Dox)-regulated form of the transcription factor Hoxb8 and a puromycin (Puro) selection cassette. Cas9-Hoxb8 cells could be kept for weeks in a non-differentiated state by culture in the presence of mSCF, huIL-11, huFlt3L, mIL-3, Dox, and Puro. Genetic modifications such as overexpression or CRISPR/Cas9-mediated knockout could be introduced by viral transduction. Successfully targeted cells were selected based on fluorescence-activated cell sorting. The longevity of Cas9-Hoxb8 cells provided the opportunity to knockout multiple genes by consecutive genetic manipulations. For differentiation into macrophages or dendritic cells (DCs), mSCF, huIL-11, huFlt3L, mIL-3, Dox, and Puro were replaced with macrophage colony-stimulating factor (M-CSF) or granulocyte-macrophage colony-stimulating factor (GM-CSF).

In this study, we expanded the benefits of CRISPR/Cas9 technology in hematopoietic progenitor cells transiently immortalized by the transcription factor Hoxb8 which enforces self-renewal and arrests differentiation (24–26, 49, 50). Specifically, we conditionally immortalized hematopoietic progenitor cells from the BM of Cas9-transgenic mice by the introduction of a Dox-regulated form of Hoxb8 giving rise to Cas9-Hoxb8 cells. These cells resemble common myeloid progenitor cells that can be stably kept in the culture for at least 16 weeks (data not shown) and thus provide superfluous time for multiple genetic modifications by, in our case, consecutive retroviral or lentiviral transduction, as demonstrated for the knockout of *Ccr7, Cxcr4*, and *Trpml1*. Moreover, overexpression of the genetically encoded Ca^2+^ sensor GCaMP6S demonstrates that cells with reporter proteins or gene overexpression can also be obtained with high efficacy. Successfully manipulated cells can be purified based on the expression of fluorescent proteins, subsequently expanded, kept in culture and cryopreserved for future projects. Therefore, the Cas9-Hoxb8 cells presented in this study offer a fast track toward genetically modified myeloid cells, circumventing problems associated with low transduction of primary cells as well as time-consuming and costly breeding to obtain multiple-gene knockout mice (depicted on Figure 12).

In our hands, Cas9-Hoxb8 cells generated from lineage-depleted BM cell retained macrophage, dendritic cell and granulocyte potential. Interestingly, they did not differentiate into cDCs and pDCs in the presence of Flt3L. The non-responsiveness of Cas9-Hoxb8 cells to Flt3L might be linked to their marginal Flt3 expression (Figure 1D). In contrast, Redecke and colleagues reported that conditionally immortalized early hematopoietic progenitor cells from crude preparations of BM cells were dependent on Flt3L and could be induced by this factor to differentiate into cDCs and pDCs (24). Moreover, the Hoxb8 cells used in that study even retained B cell and limited T cell potential (24). Likewise, Hoxb8+ hematopoietic progenitor cells transduced with Cas9 maintained their potential to differentiate into pDCs, which was exploited to investigate the role of E protein TCF4 in pDC development (25). Observed differences between these Hoxb8 cells (24, 25) and our Cas9-Hoxb8 cells most likely could be attributed to the differences in the experimental protocols used for immortalization of hematopoietic progenitor cells from BM, such as differences in viral vector design or viral particle tropism leading to the selective infection of progenitors lacking pDC potential. Similarly, the cytokine cocktail, in which the immortalized cells are raised and propagated, presumably shapes their lineage potential. The combination of mSCF, huIL-11, huFlt3L and mIL-3, which we employed here, has been described to support good proliferation of progenitor cells while shifting them toward the myeloid lineage (51) which is in line the with limited lineage potential that we observed in our study. Thus, in the future, further culture protocols employing different combinations of cytokines, such as those described by Lodish Lab (52–55), remain to be tested for a better preservation of the stemness of immortalized cells.

GM-CSF-derived CD11^+^MHCII^+^ DCs from bone marrow have been described to be phenotypically heterogeneous, as they expand from divergent hematopoietic progenitors (45). In this regard, the unlimited proliferative capacity of immortalized progenitor cells might offer new possibilities to obtain a defined population of either only monocyte-derived macrophage or exclusively conventional DC resembling cells from a GM-CSF differentiation culture. This could be possibly achieved by either sorting of defined monocyte or dendritic cell precursor populations from bone marrow as targets for viral transduction with Hoxb8 or, alternatively, by subcloning of cells after Hoxb8-mediated immortalization.

Within the scope of our study, we focused on Cas9-Hoxb8 cells as a source of genetically modified dendritic cells for the investigation of dendritic cell migration. In line with previous observations with Hoxb8-FL cells (24), GM-CSF-differentiated Cas9-Hoxb8 DCs showed the classical phenotype and T cell activation potential of GM-CSF-differentiated BM-derived DCs. Further, they entered the lymph node following intralymphatic injection in a CCR7-dependent manner in a similar fashion as described earlier for BM-derived DCs (9). In the present study, we also addressed the role of the lysosomal ion channel TRPML1 in homing of lymph-delivered DCs. A recent study reported that TRPML1 is required for persistent migration and chemotaxis of activated DCs and *Trpml1*^−/−^ mature DCs were less efficient in migrating to the draining lymph node when transferred into the footpad of recipient mice (46). To clarify whether *Trpml1*-deficient DCs are impaired in exiting from peripheral tissue or in exiting from the subcapsular sinus toward the deep T cell zone, we intralymphatically injected *Trpml1*-deficient DCs generated from GM-CSF- and LPS-stimulated Cas9-Hoxb8 cells and found that they translocated slower from the SCS to the T cell zone of the LN than *Trpml1*^+/+^ DCs.

Besides being a potent tool for investigation of gene function by their mutation, Cas9-Hoxb8 cells also provide an opportunity to investigate cellular functions by gene overexpression or expression of different reporters. Here, we have focused on chemokine-induced Ca^2+^ signaling by tracking changes in intracellular Ca^2+^ concentration in migrating DC expressing the genetically encoded Ca^2+^ sensor GCaMP6S (37). Our combination of immunoengineering approach and two-photon microscopy allowed us to gain *in vivo* insights into chemokine receptor-induced signaling cascades involved in the entry process of DCs arriving via afferent lymphatics. Intralymphatically administered BM-derived DCs transmigrate through the floor of LN subcapsular sinus in a highly directional way that depends on the interaction of CCR7 with its ligands CCL19 and CCL21 (8, 9). While long lasting Ca^2+^signals were present in 39% of GCaMP6S^+^ *Ccr7*^+/+^ Cas9-Hoxb8-DCs, they were only observed in 17% of GCaMP6S^+^ *Ccr7*^−/−^ Cas9-Hoxb8-DCs. As Ca^2+^ signals were observed in both, migrating DCs and DCs remaining sessile in the SCS for entire observation period of 2 h, we speculate that Ca^2+^ signals observed in *Ccr7*-deficient cells might arise from the recognition of other chemokines such as CXCL12 that is also present in the subcapsular sinus, or derive independent from any chemokine receptor signaling. As alterations of intracellular Ca^2+^ concentrations are involved in many DC functions, including their migration and formation of immunological synapses with T cells (15, 56), it will be crucial in future experiments to employ our GCaMP6S^+^ Cas9-Hoxb8 cells to knockout additional genes involved in various aspects of DC function including *Trpml1, Cdc42*, or *RhoA*, which are all known to contribute for DC migration (46, 57).

Altogether, the proliferative capacity and gene editing potential of Cas9-Hoxb8 cells represent a potent platform that simultaneously enables multifaceted gene editing and overexpression of genetic reporters in many different cell types, allowing, in combination with immunophysics, almost indefinite possibilities for studies of hematopoietic cell differentiation and immune cell function.

## Author contributions

SH, KW, BB, and RF designed the study. MR generated Hoxb8 cells. SH performed viral transduction, *in vitro* differentiation and flow cytometry experiments and analyzed the data. KW performed all intralymphatic injections, two-photon microscopy experiments and analyzed immunohistological data. MR, ASe, MG, AS, LL, and MP designed, cloned and validated viral vectors. AS and MG overviewed viral vector design and production. MG and MP produced viral vector supernatants. DNF performed DC-T cell co-culture experiments and analyzed the data. GEP performed gene editing efficiency analysis. KW, MP, GP, and BB analyzed two-photon microscopy data. AB helped with cell cultures and performed flow cytometry and immunofluorescent staining. BB and RF jointly supervised the project. BB and SH wrote the manuscript. All authors contributed to manuscript revision, read and approved the submitted version.

### Conflict of interest statement

The authors declare that the research was conducted in the absence of any commercial or financial relationships that could be construed as a potential conflict of interest.

## Supplementary material

Supplementary Video 1 Ca^2+^ concentration changes in *Ccr7*^+/+^ and *Ccr7*^−/−^ Hoxb8-DCs transduced with Ca^2+^ sensor GCaMP6S during entry into the lymph node. Example two-photon microscopy imaging of GCaMP6S^+^ *Ccr7*^+/+^ and *Ccr7*^−/−^ Hoxb8-DCs (green) in the subcapsular sinus of popliteal LN after intralymphatic injection into the lymph vessel draining to this LN. Scale bar represents 20 μm. Representative of 7 movies from 7 mice for each cell genotype.

## Abbreviations

BM: bone marrow
cDC: conventional dendritic cell
DC: dendritic cell
Dox: doxycycline
dTom: dTomato
GCaMP6S: GFP-Calmodulin-M13-6 slow
GM-CSF: granulocyte macrophage colony-stimulating factor
LPS: lipopolysaccharide
LN: lymph node
M-CSF: macrophage colony-stimulating factor
pDC: plasmacytoid dendritic cell
pt: post transduction
Puro: puromycin
SCS: subcapsular sinus
sgRNA: single guide RNA.

## References

[1] Worbs T Hammerschmidt SI Förster R. Dendritic cell migration in health and disease. Nat Rev Immunol. (2017) 17:30–48. 10.1038/nri.2016.116 27890914

[2] Permanyer M Bošnjak B Förster R. Dendritic cells, T cells and lymphatics: dialogues in migration and beyond. Curr Opin Immunol. (2018) 53:173–9. 10.1016/j.coi.2018.05.004 29857205

[3] Ohl L Mohaupt M Czeloth N Hintzen G Kiafard Z Zwirner J. CCR7 governs skin dendritic cell migration under inflammatory and steady-state conditions. Immunity (2004) 21:279–88. 10.1016/j.immuni.2004.06.014 15308107

[4] Weber M Hauschild R Schwarz J Moussion C de Vries I Legler DF. Interstitial dendritic cell guidance by haptotactic chemokine gradients. Science (2013) 339:328–32. 10.1126/science.1228456 23329049

[5] Vaahtomeri K Brown M Hauschild R De Vries I Leithner AF Mehling M. Locally triggered release of the chemokine CCL21 promotes dendritic cell transmigration across lymphatic endothelia. Cell Rep. (2017) 19:902–9. 10.1016/j.celrep.2017.04.027 28467903 PMC5437727

[6] Pflicke H Sixt M. Preformed portals facilitate dendritic cell entry into afferent lymphatic vessels. J Exp Med. (2009) 206:2925–35. 10.1084/jem.20091739 19995949 PMC2806476

[7] Russo E Teijeira A Vaahtomeri K Willrodt AH Bloch JS Nitschké M. Intralymphatic CCL21 promotes tissue egress of dendritic cells through afferent lymphatic vessels. Cell Rep. (2016) 14:1723–34. 10.1016/j.celrep.2016.01.048 26876174

[8] Ulvmar MH Werth K Braun A Kelay P Hub E Eller K. The atypical chemokine receptor CCRL1 shapes functional CCL21 gradients in lymph nodes. Nat Immunol. (2014) 15:623–30. 10.1038/ni.2889 24813163

[9] Braun A Worbs T Moschovakis GL Halle S Hoffmann K Bölter J. Afferent lymph-derived T cells and DCs use different chemokine receptor CCR7-dependent routes for entry into the lymph node and intranodal migration. Nat Immunol. (2011) 12:879–87. 10.1038/ni.2085 21841786

[10] Johnson LA Jackson DG. The chemokine CX3CL1 promotes trafficking of dendritic cells through inflamed lymphatics. J Cell Sci. (2013) 126:5259–70. 10.1242/jcs.135343 24006262 PMC3828594

[11] Kabashima K Shiraishi N Sugita K Mori T Onoue A Kobayashi M. CXCL12-CXCR4 engagement is required for migration of cutaneous dendritic cells. Am J Pathol. (2007) 171:1249–57. 10.2353/ajpath.2007.070225 17823289 PMC1988874

[12] Acton SE Astarita JL Malhotra D Lukacs-Kornek V Franz B Hess PR. Podoplanin-rich stromal networks induce dendritic cell motility via activation of the C-type lectin receptor CLEC-2. Immunity (2012) 37:276–89. 10.1016/j.immuni.2012.05.022 22884313 PMC3556784

[13] Johnson LA Banerji S Lawrance W Gileadi U Prota G Holder K A. Dendritic cells enter lymph vessels by hyaluronan-mediated docking to the endothelial receptor LYVE-1. Nat Immunol. (2017) 18:762–70. 10.1038/ni.3750 28504698

[14] Rathinasamy A Czeloth N Pabst O Förster R Bernhardt G. The origin and maturity of dendritic cells determine the pattern of sphingosine 1-phosphate receptors expressed and required for efficient migration. J Immunol. (2010) 185:4072–81. 10.4049/jimmunol.1000568 20826749

[15] Heuzé ML Vargas P Chabaud M Le Berre M Liu YJ Collin O. Migration of dendritic cells: physical principles, molecular mechanisms, and functional implications. Immunol Rev. (2013) 256:240–54. 10.1111/imr.12108 24117825

[16] Lämmermann T Bader BL Monkley SJ Worbs T Wedlich-Söldner R Hirsch K. Rapid leukocyte migration by integrin-independent flowing and squeezing. Nature (2008) 453:51–5. 10.1038/nature06887 18451854

[17] Wiedenheft B Sternberg SH Doudna JA. RNA-guided genetic silencing systems in bacteria and archaea. Nature (2012) 482:331–8. 10.1038/nature10886 22337052

[18] Jinek M Chylinski K Fonfara I Hauer M Doudna JA Charpentier E. A Programmable dual-RNA – guided DNA endonuclease in adaptive bacterial immunity. Science (2012) 337:816–22. 10.1126/science.1225829 22745249 PMC6286148

[19] Cong L Ran A Cox D Shuailiang L Barretto R Habib N Multiplex genome engineering using CRIPSR/Cas system. Science (2013) 339:819–24. 10.1126/science.1231143 23287718 PMC3795411

[20] Mali P Yang L Esvelt KM Aach J Guell M DiCarlo JE. RNA-guided human genome engineering via Cas9. Science (2013) 339:823–6. 10.1126/science.1232033 23287722 PMC3712628

[21] Chu VT Graf R Wirtz T Weber T Favret J Li X. Efficient CRISPR-mediated mutagenesis in primary immune cells using CrispRGold and a C57BL/6 Cas9 transgenic mouse line. Proc Natl Acad Sci USA. (2016) 113:12514–9. 10.1073/pnas.1613884113 27729526 PMC5098665

[22] Platt RJ Chen S Zhou Y Yim MJ Swiech L Kempton HR. CRISPR-Cas9 knockin mice for genome editing and cancer modeling. Cell (2014) 159:440–55. 10.1016/j.cell.2014.09.014 25263330 PMC4265475

[23] Parnas O Jovanovic M Eisenhaure TM Herbst RH Dixit A Ye CJ. A genome-wide CRISPR screen in primary immune cells to dissect regulatory networks. Cell (2015) 162:675–86. 10.1016/j.cell.2015.06.059 26189680 PMC4522370

[24] Redecke V Wu R Zhou J Finkelstein D Chaturvedi V High AA. Hematopoietic progenitor cell lines with myeloid and lymphoid potential. Nat Methods (2013) 10:795–803. 10.1038/nmeth.2510 23749299 PMC4131762

[25] Grajkowska LT Ceribelli M Lau CM Warren ME Tiniakou I Nakandakari Higa S. Isoform-specific expression and feedback regulation of E protein TCF4 control dendritic cell lineage specification. Immunity (2017) 46:65–77. 10.1016/j.immuni.2016.11.006 27986456 PMC5243153

[26] Leithner A Renkawitz J De Vries I Hauschild R Häcker H Sixt M. Fast and efficient genetic engineering of hematopoietic precursor cells for the study of dendritic cell migration. Eur J Immunol. (2018) 48:1074–7. 10.1002/eji.201747358 29436709

[27] Hope T. Improving the post-transcriptional aspects of lentiviral vectors. Curr Top Microbiol Immunol. (2002) 261:179–89. 10.1007/978-3-642-56114-6_9 11892247

[28] Schambach A Galla M Maetzig T Loew R Baum C. Improving transcriptional termination of self-inactivating gamma-retroviral and lentiviral vectors. Mol Ther. (2007) 15:1167–73. 10.1038/sj.mt.6300152 17406345

[29] Stemmer M Thumberger T Del Sol Keyer M Wittbrodt J Mateo JL. CCTop: an intuitive, flexible and reliable CRISPR/Cas9 target prediction tool. PLoS ONE (2015) 10:e0124633. 10.1371/journal.pone.0124633 25909470 PMC4409221

[30] Bošnjak B Permanyer M Sethi MK Galla M Maetzig T Heinemann D CRISPR/Cas9 genome editing using gold-nanoparticle-mediated laserporation. Adv Biosyst. (2018). 10.1002/adbi.201700184. [Epub ahead of print].

[31] Heckl D Kowalczyk MS Yudovich D Belizaire R Puram R V McConkey ME. Generation of mouse models of myeloid malignancy with combinatorial genetic lesions using CRISPR-Cas9 genome editing. Nat Biotechnol. (2014) 32:941–6. 10.1038/nbt.2951 24952903 PMC4160386

[32] Heinz N Schambach A Galla M Maetzig T Baum C Loew R. Retroviral and transposon-based tet-regulated all-in-one vectors with reduced background expression and improved dynamic range. Hum Gene Ther. (2011) 22:166–76. 10.1089/hum.2010.099 20825282

[33] Dreyer AK Hoffmann D Lachmann N Ackermann M Steinemann D Timm B. TALEN-mediated functional correction of X-linked chronic granulomatous disease in patient-derived induced pluripotent stem cells. Biomaterials (2015) 69:191–200. 10.1016/j.biomaterials.2015.07.057 26295532

[34] Warlich E Kuehle J Cantz T Brugman MH Maetzig T Galla M. Lentiviral vector design and imaging approaches to visualize the early stages of cellular reprogramming. Mol Ther. (2011) 19:782–9. 10.1038/mt.2010.314 21285961 PMC3070104

[35] Schambach A Bohne J Chandra S Will E Margison GP Williams DA. Equal potency of gammaretroviral and lentiviral SIN vectors for expression of O6-methylguanine-DNA methyltransferase in hematopoietic cells. Mol Ther. (2006) 13:391–400. 10.1016/j.ymthe.2005.08.012 16226060

[36] Morita S Kojima T Kitamura T. Plat-E: an efficient and stable system for transient packaging of retroviruses. Gene Ther. (2000) 7:1063–6. 10.1038/sj.gt.3301206 10871756

[37] Chen TW Wardill TJ Sun Y Pulver SR Renninger SL Baohan A. Ultrasensitive fluorescent proteins for imaging neuronal activity. Nature (2013) 499:295–300. 10.1038/nature12354 23868258 PMC3777791

[38] Halle S Keyser KA Stahl FR Busche A Marquardt A Zheng X. *In vivo* killing capacity of cytotoxic T cells is limited and involves dynamic interactions and T cell cooperativity. Immunity (2016) 44:233–45. 10.1016/j.immuni.2016.01.010 26872694 PMC4846978

[39] Galla M Schambach A Baum C. Retrovirus-based mRNA transfer for transient cell manipulation. Methods Mol Biol. (2013) 969:139–61. 10.1007/978-1-62703-260-5_10 23296933

[40] Rothe M Rittelmeyer I Iken M Rüdrich U Schambach A Glage S. Epidermal growth factor improves lentivirus vector gene transfer into primary mouse hepatocytes. Gene Ther. (2012) 19:425–34. 10.1038/gt.2011.117 21850050

[41] Ho VWH Sly LM. Derivation and characterization of murine alternatively activated (M2) macrophages. Methods Mol Biol. (2009) 531:173–85. 10.1007/978-1-59745-396-7_12 19347318

[42] Kohli K Janssen A Förster R Plasmacytoid dendritic cells induce tolerance predominantly by cargoing antigen to lymph nodes. Eur J Immunol. (2016) 58:7250–7. 10.1002/eji.201646359 PMC5129535 27592607

[43] Hsiau T Maures T Waite K Yang J Kelso R Holden K Inference of CRISPR edits from sanger trace data. bioRxiv [preprint]. (2018) 10.1101/251082 35119294

[44] Halle S Dujardin HC Bakocevic N Fleige H Danzer H Willenzon S. Induced bronchus-associated lymphoid tissue serves as a general priming site for T cells and is maintained by dendritic cells. J Exp Med. (2009) 206:2593–601. 10.1084/jem.20091472 19917776 PMC2806625

[45] Helft J Böttcher J Chakravarty P Zelenay S Huotari J Schraml BU. GM-CSF mouse bone marrow cultures comprise a heterogeneous population of CD11c(+)MHCII(+) macrophages and dendritic cells. Immunity (2015) 42:1197–211. 10.1016/j.immuni.2015.05.018 26084029

[46] Bretou M Sáez PJ Sanséau D Maurin M Lankar D Chabaud M. Lysosome signaling controls the migration of dendritic cells. Sci Immunol. (2017) 2:eaak9573. 10.1126/sciimmunol.aak9573 29079589

[47] Noser JA Towers GJ Sakuma R Dumont J-M Collins MKL Ikeda Y. Cyclosporine increases human immunodeficiency virus type 1 vector transduction of primary mouse cells. J Virol. (2006) 80:7769–74. 10.1128/JVI.02427-05 16840358 PMC1563702

[48] Geis FK Galla M Hoffmann D Kuehle J Zychlinski D Maetzig T Potent and reversible lentiviral vector restriction in murine induced pluripotent stem cells. Retrovirology (2017) 14:34 10.1186/s12977-017-0358-1 28569216 PMC5452410

[49] Rosas M Osorio F Robinson MJ Davies LC Dierkes N Jones SA. Hoxb8 conditionally immortalised macrophage lines model inflammatory monocytic cells with important similarity to dendritic cells. Eur J Immunol. (2011) 41:356–65. 10.1002/eji.201040962 21268006

[50] Wang GG Calvo KR Pasillas MP Sykes DB Häcker H Kamps MP. Quantitative production of macrophages or neutrophils *ex vivo* using conditional Hoxb8. Nat Methods (2006) 3:287–93. 10.1038/nmeth865 16554834

[51] Li Z Schwieger M Lange C Kraunus J Sun H van den Akker E. Predictable and efficient retroviral gene transfer into murine bone marrow repopulating cells using a defined vector dose. Exp Hematol. (2003) 31:1206–14. 10.1016/j.exphem.2003.08.008 14662326

[52] Zhang CC Lodish HF. Murine hematopoietic stem cells change their surface phenotype during *ex vivo* expansion. Blood (2005) 105:4314–20. 10.1182/blood-2004-11-4418 15701724 PMC1895041

[53] Zhang CC Kaba M Iizuka S Huynh H Lodish HF. Angiopoietin-like 5 and IGFBP2 stimulate *ex vivo* expansion of human cord blood hematopoietic stem cells as assayed by NOD/SCID transplantation. Blood (2008) 111:3415–23. 10.1182/blood-2007-11-122119 18202223 PMC2275010

[54] Zhang CC Lodish HF. Cytokines regulating hematopoietic stem cell function. Curr Opin Hematol. (2008) 15:307–11. 10.1097/MOH.0b013e3283007db5 18536567 PMC2677548

[55] Zhang CC Kaba M Ge G Xie K Tong W Hug C. Angiopoietin-like proteins stimulate *ex vivo* expansion of hematopoietic stem cells. Nat Med. (2006) 12:240–5. 10.1038/nm1342 16429146 PMC2771412

[56] Shumilina E Huber SM Lang F. Ca2+ signaling in the regulation of dendritic cell functions. Am J Physiol Cell Physiol. (2011) 300:C1205–14. 10.1152/ajpcell.00039.2011 21451105

[57] Vargas P Maiuri P Bretou M Sáez PJ Pierobon P Maurin M Innate control of actin nucleation determines two distinct migration behaviours in dendritic cells. Nat Cell Biol. (2016) 18:43–53. 10.1038/ncb3284 26641718 PMC5885286

